# Patients’ knowledge, preferences, and perspectives about data protection and data control: an exploratory survey

**DOI:** 10.3389/fphar.2023.1280173

**Published:** 2024-02-20

**Authors:** Teodora Lalova-Spinks, Robbe Saesen, Mitchell Silva, Jan Geissler, Iryna Shakhnenko, Jennifer Catherine Camaradou, Isabelle Huys

**Affiliations:** ^1^ Clinical Pharmacology and Pharmacotherapy, Department of Pharmaceutical and Pharmacological Sciences, KU Leuven, Leuven, Belgium; ^2^ Center for IT & IP Law (CiTiP), KU Leuven, Leuven, Belgium; ^3^ European Organisation for Research and Treatment of Cancer (EORTC), Brussels, Belgium; ^4^ EUPATI Belgium vzw, Brussels, Belgium; ^5^ Patvocates, Munich, Germany; ^6^ Individual Patient Expert, Exeter, Devon, United Kingdom

**Keywords:** GDPR, patient empowerment, data control, data altruism, health research, clinical trials, Data Governance Act, European Health Data Space

## Abstract

**Background:** In the European Union, the General Data Protection Regulation (GDPR) plays a central role in the complex health research legal framework. It aims to protect the fundamental right to the protection of individuals’ personal data, while allowing the free movement of such data. However, it has been criticized for challenging the conduct of research. Existing scholarship has paid little attention to the experiences and views of the patient community. The aim of the study was to investigate 1) the awareness and knowledge of patients, carers, and members of patient organizations about the General Data Protection Regulation, 2) their experience with exercising data subject rights, and 3) their understanding of the notion of “data control” and preferences towards various data control tools.

**Methods:** An online survey was disseminated between December 2022 and March 2023. Quantitative data was analyzed descriptively and inferentially. Answers to open-ended questions were analyzed using the thematic analysis method.

**Results:** In total, 220 individuals from 28 European countries participated. The majority were patients (77%). Most participants had previously heard about the GDPR (90%) but had not exercised any of their data subject rights. Individual data control tools appeared to be marginally more important than collective tools. The willingness of participants to share personal data with data altruism organizations increased if patient representatives would be involved in the decision-making processes of such organizations.

**Conclusion:** The results highlighted the importance of providing in-depth education about data protection. Although participants showed a slight preference towards individual control tools, the reflection based on existing scholarship identified that individual control holds risks that could be mitigated through carefully operationalized collective tools. The discussion of results was used to provide a critical view into the proposed European Health Data Space, which has yet to find a productive balance between individual control and allowing the reuse of personal data for research.

## 1 Introduction

### 1.1 A complex EU legal framework for health research and use of personal data

**FIGURE 1:**
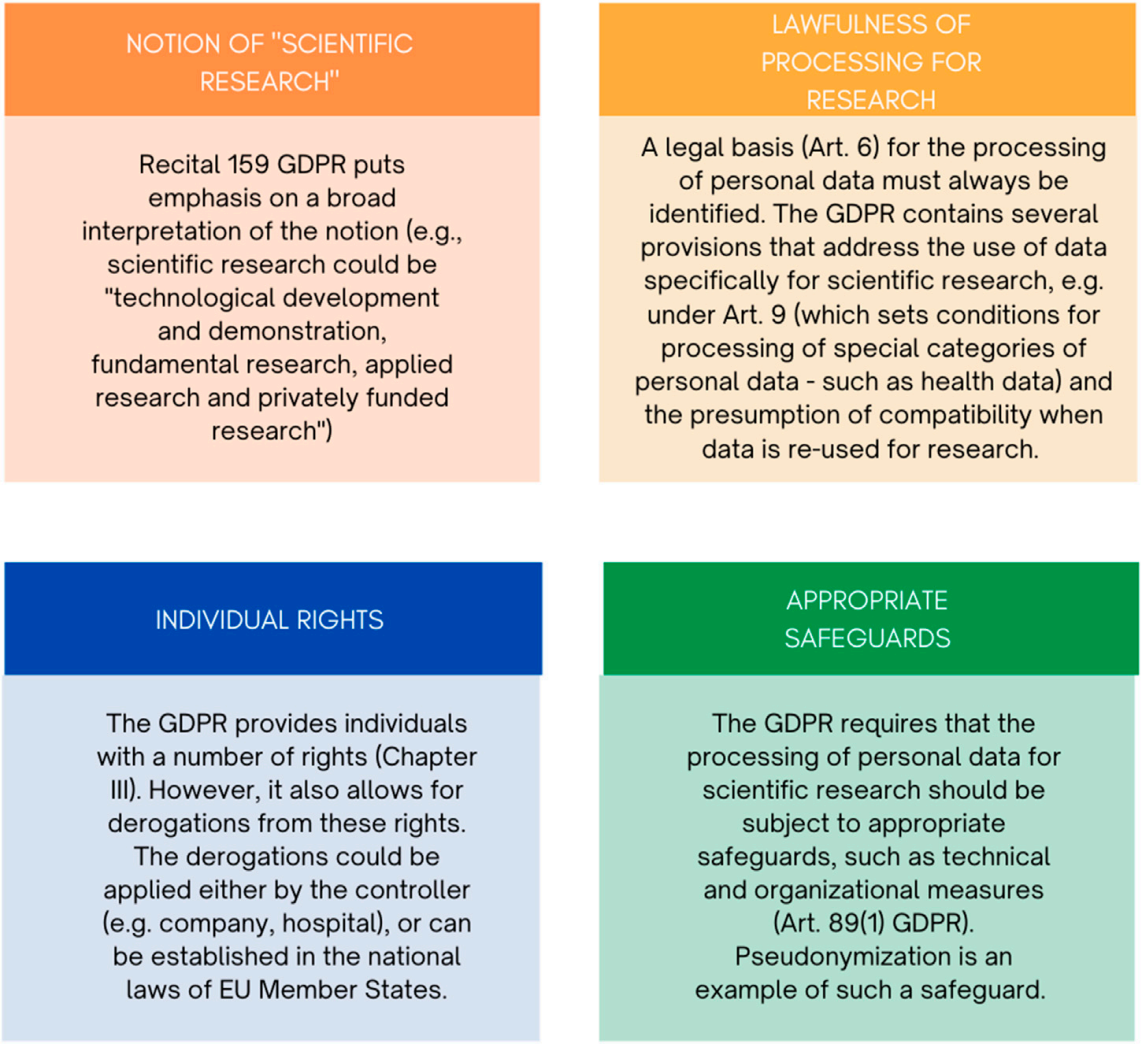
Building elements of the scientific research regime in the GDPR.

Health research relies on patients’ participation and the use and reuse of their personal, health-related data. In the European Union (EU), the legal and ethical framework for health research is complex and highly divergent (Lalova et al., 2020; Hansen et al., 2021).^1^ In addition to the many specific rules governing the conduct of research, a central role is played by the EU General Data Protection Regulation (GDPR). The GDPR serves a two-fold aim: on one side, to protect individuals’ fundamental rights and freedoms, particularly the right to protection of their personal data, and on the other side, to prevent divergences hampering the free movement of personal data within the EU. Moreover, it established a legal regime for the processing of personal data for scientific research (i.e., a scientific research regime, as described by Slokenberga (Slokenberga, 2022)), which rests upon several building blocks, namely: the notion of “scientific research”, individual rights, lawfulness of the data processing and appropriate safeguards (Figure 1).^2^

However, the implementation and interpretation of the GDPR at national level is fragmented, which makes cross-border cooperation for research challenging (Lalova-Spinks et al., 2022). Scholars and practitioners have criticized the regulation for disrupting clinical trials (European Organisation for Research and Treatment of Cancer, 2020), for making the secondary use of data more difficult (Peloquin et al., 2020), and for eroding the level of protection for research participants (known as “data subjects” under the GDPR) (Staunton et al., 2019).

### 1.2 Opportunity to address the challenges

The European Commission is currently working on several legislative initiatives that have the potential to create new opportunities, but also to further complicate the legal framework for health research. These changes – part of the European Strategy for Data – aim to ensure that more data is available for use in the economy and society, while maintaining control by the individuals and companies who generate the data (European Commission, 2020). Two of the new laws deserve particular attention due to the complex ways they will interplay with the data protection legal framework.

The first is the Data Governance Act (DGA, adopted in May 2022),^3^ which aims to facilitate the reuse of certain categories of data held by public sector bodies, increase trust in data intermediation services and promote data altruism across the EU.^4^ Data altruism is defined as the voluntary sharing of personal data (based on the consent of individuals) or non-personal data (based on the permission of individuals or legal entities), without compensation and for purposes of general interest, such as healthcare and scientific research.^5^ Consents and permissions will be collected and managed by data altruism organizations registered and recognized in the EU.^6^ Such organizations will be able to process the altruistically shared data themselves, or to make it available for use by other data users (natural or legal persons).^7^

The second legislative initiative of particular interest is the proposal for a European Health Data Space (EHDS) regulation, put forward by the European Commission in May 2022. The EHDS aims to 1) increase individuals’ control over their electronic health data, 2) create a legal framework consisting of a trusted governance mechanisms and a secure processing environment, and 3) contribute to a genuine single market for digital health products and services.^8^ The EHDS proposal builds on existing relevant legislation, *inter alia*, the GDPR and the DGA. It sets down rules for the primary and secondary use of data. While primary use focuses on the governance of data processing in relation to the provision of healthcare and elaborates rights and mechanisms that complement the data subject rights under the GDPR,^9^ the rules on secondary use aim to create a permit-based system that allows the sharing of health data for a vast set of specific purposes, including scientific research, but also, e.g., training and the evaluation of algorithms.^10^ By submitting a single authorization request to one of the new so-called health data access bodies (HDAB), data users interested in conducting international health studies will be able to access patient data from various Member States, without having to comply with the divergent national laws governing access to data. The secondary use provisions in the EHDS proposal are also the first ones to include sector-specific rules on data altruism. In essence, the EHDS puts forward a second scientific research regime, seen as complementary to the one in the GDPR, and promising to address the existing challenges for health research (Slokenberga, 2022).

### 1.3 A growing focus on patient empowerment

The ongoing (r)evolution in the legal framework could be linked to the Europe-wide trend towards a patient-focused approach in health research, in which the concept of patient empowerment holds center stage (Verhenneman, 2021). Patient empowerment (also referred to in literature as engagement or involvement), aims to ensure that patients’ needs and priorities in healthcare and research are identified, met and are used to facilitate a proactive attitude of patients in the management of their health across various phases of their patient journey (Hoos et al., 2015). Involvement can occur at all stages of the research and development cycle (e.g., setting research priorities, research design, research conduct, post-approval, and communication) (Geissler et al., 2017). It can take various forms, such as patient advisory panels in academic institutions and pharmaceutical companies or patient representatives as members in some of the scientific committees or working groups of regulatory authorities (European Medicines Agency and national bodies) (Geissler et al., 2017). Growing evidence suggests that patient empowerment provides value for all stakeholders, whether they are patients, researchers, industry, regulatory bodies, or policymakers (Phillips et al., 2014; Hoos et al., 2015; Parsons et al., 2015; Supple et al., 2015; Wicks et al., 2015). The potential benefits have been reported to include identifying more relevant research priorities, patient-relevant research methods and findings, and therapies better targeted at patients’ needs (Geissler et al., 2017). It has also been discussed that patient empowerment may help to improve recruitment and retention in clinical trials (Donovan et al., 2002; Wicks et al., 2015). The importance of patient empowerment has started to become recognized in international instruments. For instance, the revised International ethical guidelines for health-related research involving humans, prepared by the Council for International Harmonization of Medical Sciences (CIOMS) contain provisions on community engagement. Namely, researchers, sponsors, and health authorities are advised to engage participants and communities in a meaningful way in the research and the dissemination of research results.^11^

### 1.4 Data control

The growth of patient empowerment since the 1970s could be compared to and is intertwined with the evolution of the data protection legal framework, and the concept of data control in particular (Verhenneman, 2021). The notion of data control is central to the fundamental right to data protection (Ausloos, 2020) and to the GDPR,^12^ but a clear definition of it does not exist at present (Ducuing et al., 2021). Data control (i.e., the control over one’s own personal data) plays a crucial role for overcoming the increasing power asymmetries between the entities (in all sectors, including health) that use data and the individuals whose data is used (Ausloos, 2020). According to the European Commission, control can be achieved through “tools to decide at a granular level about what is done with [the individual’s personal] data” (European Commission, 2020). Such tools could be understood to be individual or collective.

Examples of individual control tools from a legal perspective could be given at the two main stages of exercising control in health research: 1) data collection, i.e., prior to the start of a study, and 2) after personal data has been collected, i.e., during the conduct of a study and subsequent possible data reuse (Vayena and Blasimme, 2017; Purtova, 2014; Article 29 Working Party, 2018). Related to the first stage (data collection), historically much emphasis has been placed on consent as a valid legal basis^13^ for the processing of personal data (Verhenneman, 2021). Related to the second stage (post data collection), emphasis is put on the data subject rights provided by the GDPR^14^ (Table 1). These rights impose positive obligations on data controllers (i.e., the organizations or individuals who process the data, such as pharmaceutical companies or hospitals). Transparency rights^15^ are also important for enabling control in both stages (Naudts et al., 2022), because a prerequisite for exercising one’s data subject rights or providing valid consent is being informed about the data processing in the first place. Individual control could be linked to the empowerment of the patient in their subjective interest to manage the use of their personal data and be offered sufficient protection of their fundamental right to data protection.

**TABLE 1:** Overview of data subject rights under the GDPR.

1. Right to withdraw consent	2. Right to access	3. Right to rectification	4. Right to erasure
If personal data is processed on the legal basis of consent, the data subject has the right to withdraw their consent at any time. Withdrawing should be as easy as giving consent. (Art. 7(3))	The right of the data subject to obtain 1) confirmation whether personal data about themselves is processed, 2) information about the processing, and 3) copy of the personal data. (Art. 15)	The right of the data subject to change data that they believe is inaccurate or out-of-date, as well as to supplement incomplete data. (Art. 16)	The right of the data subject to request that their personal data is deleted if certain circumstances apply. (Art. 17)

5. Right to restriction	6. Right to data portability	7. Right to object	8. Right in the context of automated decision-making
Data subjects have the right to restrict the processing of their personal data under certain circumstances. (Art. 18)	The right of data subjects to receive their personal data in a structured, commonly used, and machine-readable format, and to transfer it or request for it to be transferred to another data controller, if technically feasible. (Art. 20)	Data subjects have the right to object to the processing of their personal data in certain circumstances. (Art. 21)	Data subjects have the right to not be subject to a decision based solely on automated decision-making which has legal effects concerning them. (Art.22)

In addition to individual control tools, the importance of collective control tools is increasingly discussed in literature, both in a sector-agnostic context (Mahieu and Ausloos, 2020; Rerolle and Roussoulieres, 2022), and in the specific case of health research (Kickbusch et al., 2021). One example of a collective tool would be enriching individual tools with collective actions. For instance, Article 80(1) GDPR provides a specific role for not-for-profit organizations by affording them the right to make complaints and litigate in the name of data subjects, but this tool is underused at the moment (Mahieu and Ausloos, 2020). The new legislative acts (such as the DGA and the EHDS proposal) also establish control tools which could be seen as collective ones. For instance, the DGA puts forward a special category of data intermediation services which seek to enhance the influence of individuals by assisting them in exercising their data subject rights.^16^ It also provides for the establishment of data cooperatives which will aim to strengthen the position of individuals in, e.g., making informed choices before consenting to data use.^17^ The data altruism mechanism, established by the DGA, could be seen as another example, as data altruism organizations would effectively exercise control on behalf of the altruistic individuals. Collective control tools can be associated with patient empowerment at a community level and could help increasing the overall willingness of patients to share their personal data for research.

### 1.5 The research gap

With respect to patient empowerment in a broad sense, sociomedical research has progressively recognized the importance of studying the views of patients and caregivers, finding out what matters most to them in terms of their disease or treatments, and including them as equal partners in co-designing research. An example pertains to patient preference studies which seek to investigate what treatment characteristics (called “attributes”) matter to patients, with the goal of informing decision-making and studies revealing patient perspectives on unmet medical needs (Janssens et al., 2022; The PREFER consortium, 2022). However, when it comes to the topic of data control, the patients’ voice is predominantly missing, despite the fact that control is promoted as a key policy objective (European Commission, 2020). Furthermore, empirical studies on the application of the data protection legal framework in the field of health research, are still largely lacking (Ienca et al., 2019), although examples are beginning to emerge (e.g., Lalova-Spinks et al., 2022; McLennan et al., 2022) While citizens’ and consumers’ awareness of the GDPR and attitudes toward data sharing in a broader sense have been investigated in the past (Courbier et al., 2019; Broes et al., 2020; Strycharz et al., 2020; Johansson et al., 2021), patients’ knowledge and perspectives on data control have not yet been elucidated. The European Commission is making steps towards remedying this gap with the Towards European Health Data Space (TEHDAS) project (2021–2023). TEHDAS is including EU citizens in a dialogue about the use of health data but does not systematically work on untangling the complexities related to GDPR awareness and data control (Menager et al., 2023).

The present contribution aims to fill an important gap in literature by investigating 1) the awareness and knowledge of patients, carers, and members of patient organizations about the GDPR, 2) their experience with exercising data subject rights, and 3) their understanding of the notion of “data control” and preferences towards various data control tools.

## 2 Materials and methods

### 2.1 Study design

An online survey was created and made available in English via the Qualtrics platform. It included multiple choice questions, Likert-scale questions, and open-ended questions. It consisted of two main sections: 1) Introductory questions, 2) Part A: Data protection and data control (the questionnaire is included in Supplementary material 1).^18^

Part A gathered input related to the topics of 1) GDPR awareness, 2) data subject’s rights, 3) data control, and 4) data altruism. Two GDPR knowledge-testing questions were included.^19^ First, participants had to answer who they thought was responsible for the protection of personal data when the data is used for medical research. Possible options included the individual or entity using the personal data (i.e., the data controller or processor in GDPR terms, such as a pharmaceutical company which sponsors a clinical trial), but also options that were not correct in the strict sense, such as the data protection authority, a patient organization active in the disease area under investigation, or the patient whose personal data is used. Next, participants were asked to select what rights are guaranteed under the GDPR, and the list of response options provided both existing data subject rights, and rights that the law does not envisage. Knowledge-testing questions and questions related to data subject rights were modelled following the example provided in a study about individual knowledge, reactions to and rights exercised under the GDPR in the Netherlands, conducted by Strycharz et al. (2020).

The survey questions were reviewed by and discussed with experienced patient representatives and a professor in regulatory sciences with extensive expertise in empirical and interdisciplinary research (IH). In addition, the questionnaire was pilot tested by six volunteering patient fellows of the European Patients’ Academy on Therapeutic Innovation (EUPATI). Pilot testers were recruited through EUPATIConnect, a matchmaking tool that provides the opportunity for patient experts and researchers to connect and collaborate.^20^ The study was approved by the Ethics Committee Research UZ/KU Leuven, file number S66701.

### 2.2 Participants and recruitment

The survey targeted 1) patients, 2) carers, and 3) members of patient organizations who are neither patients nor carers. To participate, respondents needed to be at least 18 years old and had to currently reside in any of the 27 EU Member States, the United Kingdom, or in the European Economic Area (EEA) countries (Switzerland, Norway, Iceland, Liechtenstein).

The survey was broadly disseminated between December 2022 and March 2023 with the help of international networks and organizations, such as the Workgroup of European Cancer Patient Advocacy Networks (WECAN), Patvocates, the European Patients’ Academy on Therapeutic Innovation (EUPATI), the European Association of Health Law (EAHL), the European Organization for Research and Treatment of Cancer (EORTC), the European Patients’ Forum (EPF) and others. It was also shared through the professional networks of the research team, and on social media. Paid advertising campaigns on Facebook and LinkedIn were activated during the dissemination period.

### 2.3 Analysis

Survey data was analyzed descriptively in Excel^®^ and inferentially using IBM^®^ SPSS^®^ Statistics 28.0. Percentages were calculated based on the number of respondents for each question. The sample size varied throughout the survey due to use of the display logic function^21^ for some questions and due to respondents’ drop-out. Measurement levels for Likert-type questions were converted to numbers (e.g., “very unlikely” corresponds to 1, “not likely” to 2, “neutral” to 3, “likely” to 4 and “very likely” to 5), allowing for the possibility to determine medians and interquartile ranges (IQRs, hereinafter represented by their lower and upper boundaries). Wilcoxon signed-rank tests were also undertaken to see if the medians leaned significantly towards either end of the answer scale.

Qualitative data (answers to open-ended questions) was analyzed thematically (Braun and Clarke, 2006), using the NVivo^®^ software. The coding of all data was performed by one researcher (TL-S), based on a working analytical framework. Prior to this, the same researcher thoroughly familiarized herself with the answers provided by all participants by rereading the completed questionnaires. Each quote included in this paper is followed by a codename, which consists of a reference to the stakeholder group to which the participant belonged, and a number, e.g., P1, C3, MPO5 (see Table 2).

**TABLE 2:** Demographic characteristics of the survey participants and codes of the stakeholder groups to which they belonged.

Stakeholder group	Code of the stakeholder group	Number of survey participants	Percentage of the total number of survey respondents
Patients	P	169 (Of whom 109 were also members of patient organizations)	77%
Carers	C	35 (Of whom 20 were also members of patient organizations)	16%
Members of patient organizations who are neither patients nor carers	MPO	16	7%

Gender representation			
Female		145	66%
Male		71	32%
Non-binary		3	1%
Preferred not to say		1	1%

Age			
18-24		2	1%
25-34		29	13%
35-44		35	16%
45-54		55	25%
55-64		53	24%
65-74		30	14%
75 or older		16	7%

Highest level of completed education			
Primary education		3	1%
Secondary education		24	11%
Bachelor's degree or equivalent		53	24%
Master's degree or equivalent		96	44%
Doctorate degree		44	20%

Total number of participants: N=220

## 3 Results

### 3.1 Demographic characteristics of the survey participants

In total, 220 individuals participated in the survey, most of whom were patients (77%, n = 169/220), see Table 2. Respondents were predominantly female (66%, n = 145/220) and above 45 years old (70%, n = 154/220). The carers in our sample took care of patients of diverse ages (e.g., children, young adults, and adults).

**FIGURE 2:**
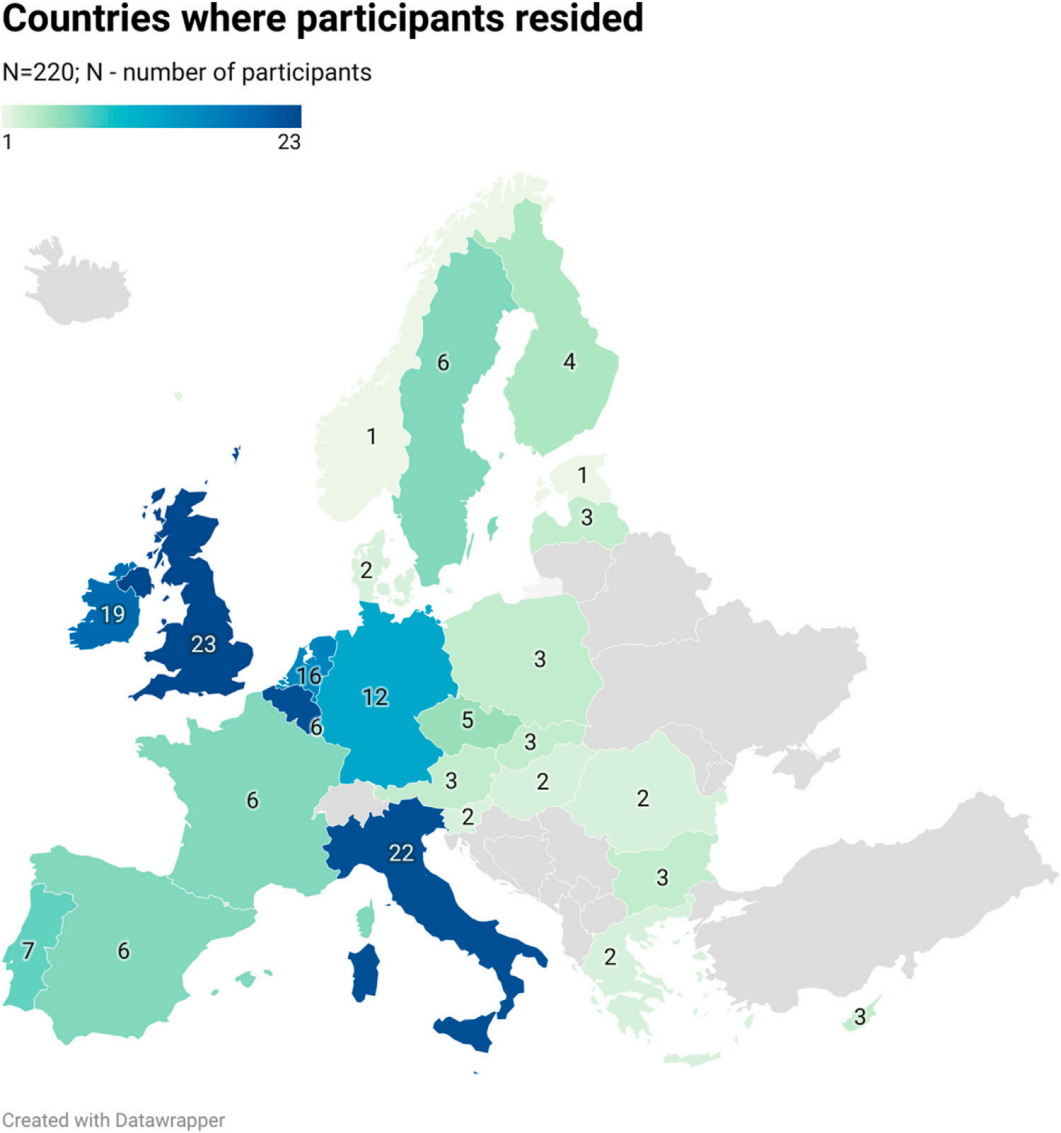
Countries where participants resided.

Out of the targeted 32 countries, 28 were represented in the survey (Figure 2). The majority of participants were based in Western and Northern Europe (32%, n = 71/220% and 31%, n = 67/220 respectively). The countries with the highest interest in the survey were the United Kingdom, Belgium, Italy, Malta, and Ireland.

**FIGURE 3:**
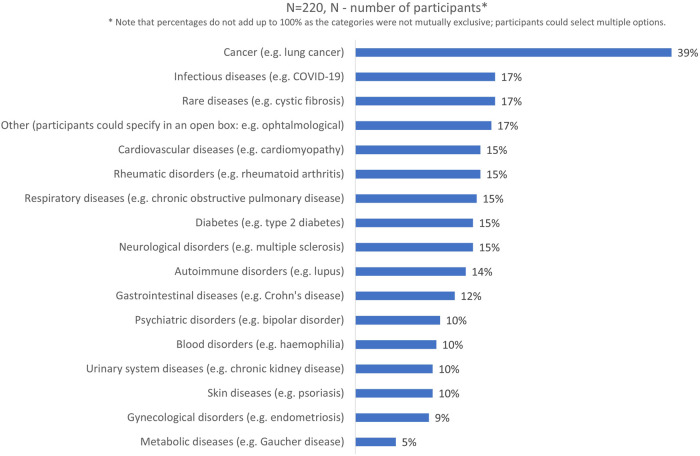
Disease areas which participants had most experience with.

The highest level of completed education for almost half of the participants was a master’s degree or equivalent (44%, n = 96/220). More than half (62%, n = 137/220) had not participated in a clinical trial, but had allowed (or knew someone who had allowed) their personal data collected in the context of healthcare to be reused for medical research (60%, n = 133/220). Participants had most experience in the areas of cancer, infectious diseases, and rare diseases (Figure 3). The majority of patients and carers were members of patient organizations (64%, n = 109/169% and 70%, n = 20/35 respectively). More than half of all respondents had previously followed courses on the topics of medicines development, clinical research, or patient engagement (64%, n = 141/220), and out of those who had received such specialized training, the majority were members of patient organizations (77%, n = 109/141).

### 3.2 Awareness about the GDPR

**FIGURE 4:**
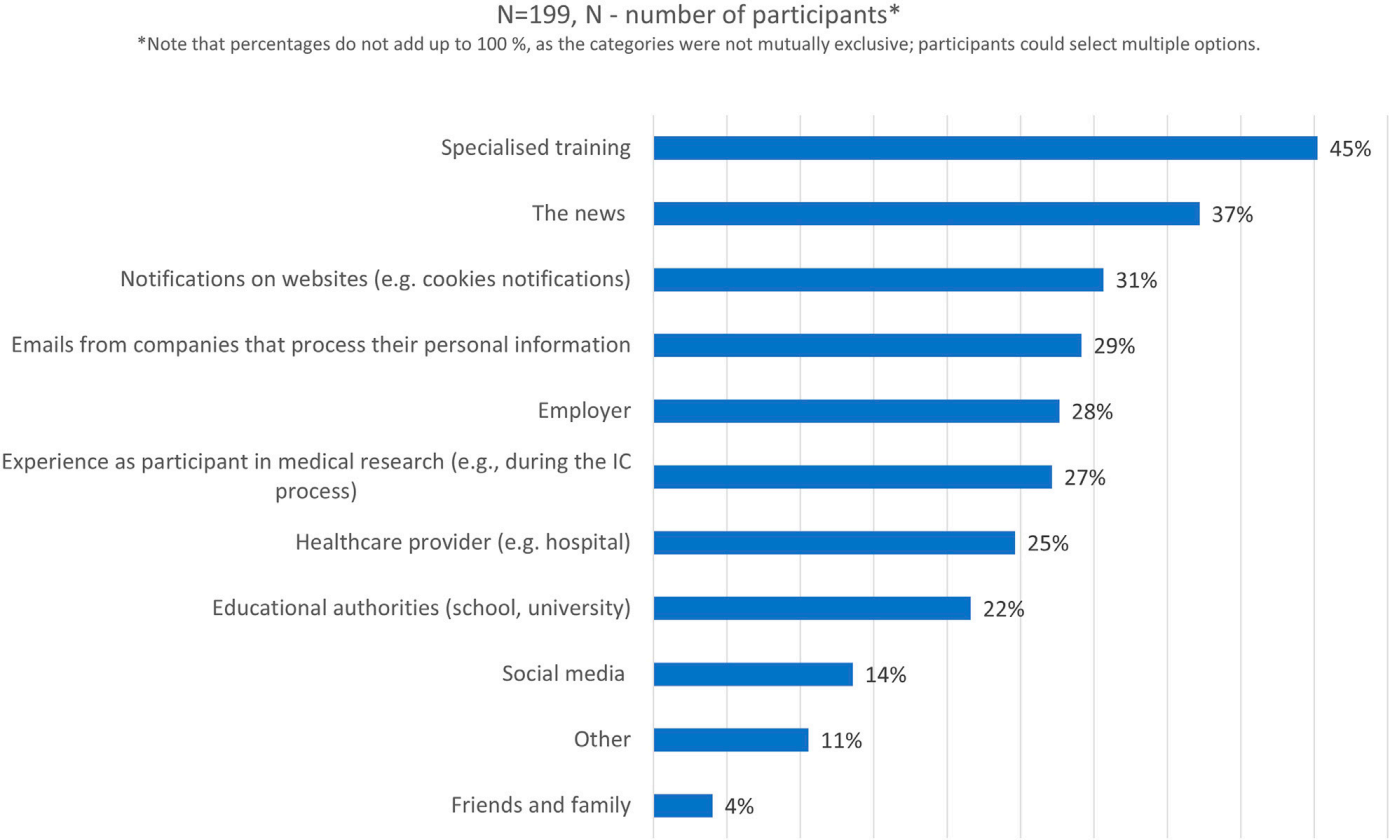
Participants’ main source of information about the GDPR.

Almost all survey respondents had previously heard about the GDPR (90%, n = 199/220). Their main source of information about it had mostly been specialized training (45%, n = 90/199) or the news (37%, n = 74/199), Figure 4. Around one-fourth (27%, n = 54/199) of the participants chose the option “experience as participant in medical research” as their main source of information about the GDPR.

Respondents mostly answered correctly the two questions that aimed to assess their GDPR knowledge. A large proportion of respondents (80%, n = 175/220) identified correctly that the responsibility for the protection of personal data when the data is used for medical research is for the individual or entity that uses the data. Nevertheless, incorrect answers were still given by many respondents, e.g., “the data protection authority” (51%, n = 113/220), or “the research ethics committee that approved the study” (28%, n = 62/220).

With respect to the list of data subject rights from which participants had to select ones that are afforded under the GDPR, only a few (10%, n = 22/220) chose the non-existent rights.

### 3.3 Data subject rights

The questions related to exercising data subject rights were formulated based on the background of the respondents and the display logic function was used to lead individuals to different questions based on their previously reported experience. Individuals who had reported experience with participation in a clinical trial or another medical study were presented with a question about whether they have exercised their rights in this particular context in the past 2 years. Respondents without experience in clinical trials or medical studies had to respond in the context of receiving care or using online health applications.

**FIGURE 5:**
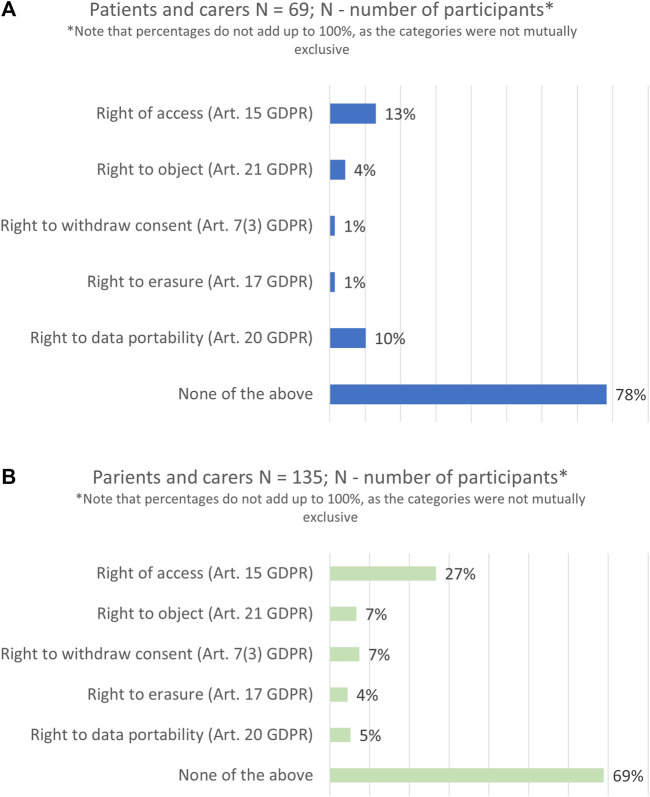
Data subject rights exercised in the past 2 years: in a research context **(A)** and in a healthcare context **(B)**.

The majority of survey respondents had never exercised the rights envisaged under the GDPR neither in research (78%, n = 54/69), nor in a care context (69%, n = 93/135), see Figure 5. Among those who had used their data subject rights, the right of access was exercised the most (13%, n = 9/69), followed by the right to data portability (10%, n = 7/69), whereas the right to erasure was used the least (1%, n = 1/69).

The study also explored whether participants planned to exercise their data subject rights in the next 1 year following the survey. In comparison to the past, participants showed a higher interest in using all rights in the future. On average the percentages almost doubled, and particularly with regards to the right of access (37%, n = 16/43).

**FIGURE 6:**
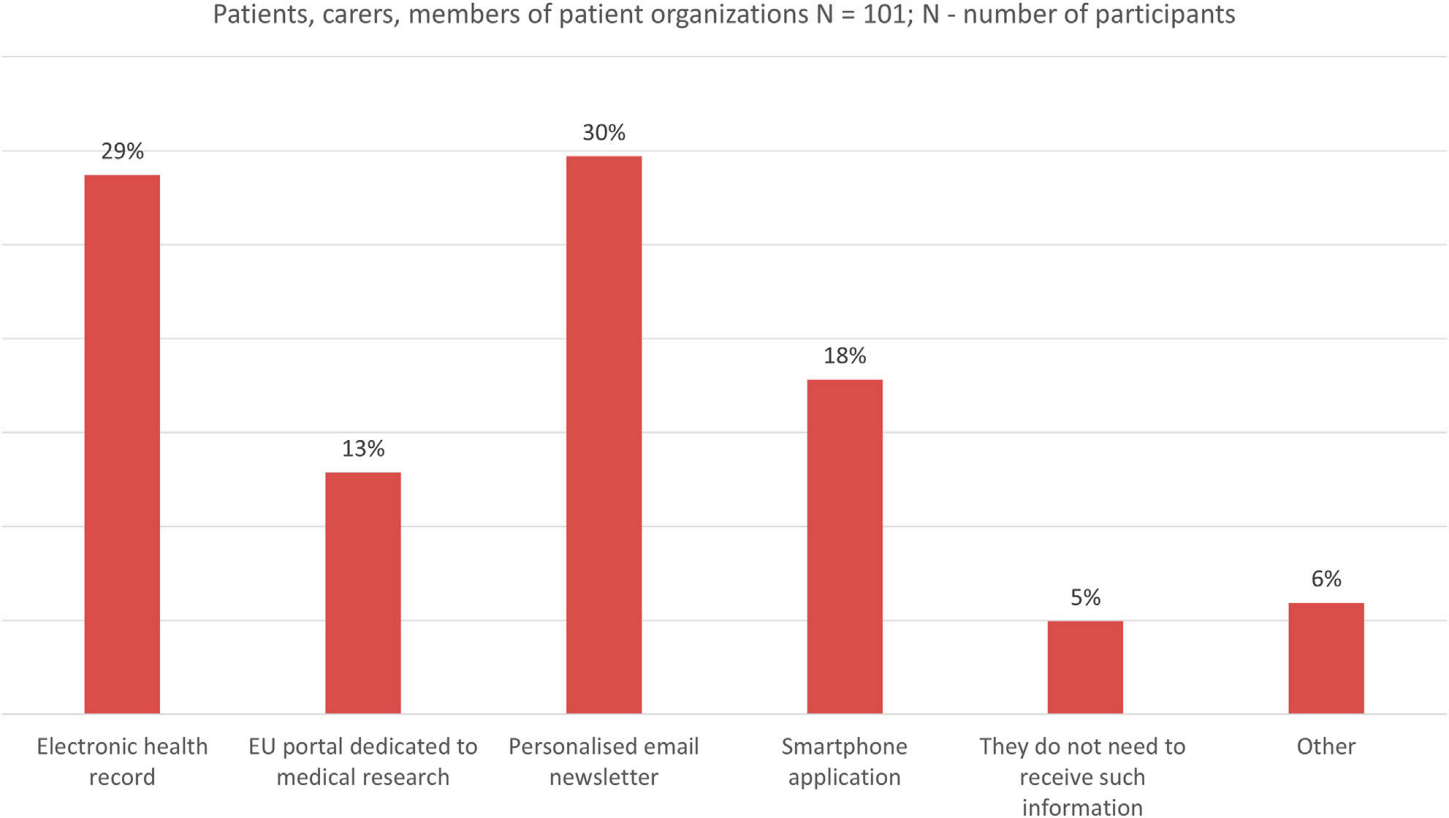
Ideal way to be informed about the use of personal data in medical research.

Finally, all survey participants were asked about their preferences (or the preference of the patient they take care of/patients they know) on ideal ways to be informed about the use of their personal data in medical research. The two most preferred options for all stakeholder groups were through a personalized email newsletter and via their electronic health record (Figure 6). Very few respondents (5%, n = 5/101) shared that they do not want to receive such information.

### 3.4 Data altruism

Participants were presented with the definition of data altruism as established under the DGA (Table 3) and were asked to indicate on a 5-point scale, ranging from “very unlikely” to “very likely”, their willingness to share personal data with data altruism organizations. In a follow-up question, they were faced with a modified definition and asked to imagine a strengthened role for patient representatives in the decision-making process of such data altruism organizations (for the definitions used in the survey, see Table 3). The involvement of patient representatives largely increased the willingness of respondents to share data. Namely, with respect to data altruism with patient involvement, participants most often selected that they are “likely” (median score of 4, IQR: 2–4.5) to share their data, whereas they were mostly “neutral” (median score of 3, IQR: 4–2) when it came to sharing data with data altruism organizations as currently envisaged under the law. There was a statistically significant difference in respondents’ answers to the two questions (*p*-value <.00001).

**TABLE 3:** Definitions of data altruism presented to survey participants.

Term	Description in the survey
Data altruism without patient involvement*	Imagine that you can consent that your personal data is shared with a so-called data altruism organization. This data altruism organization will, in turn, have the right to share your data with other users (individual researchers, companies, public bodies, etc) as long as these users intend to use the data for medical research. You can withdraw your consent at any time.
*This scenario was based on the mechanism currently established with the Data Governance Act.	
Data altruism with patient involvement	Imagine the same scenario as described above, but in this case, there are patient representatives involved in the data altruism organization. Each time the data altruism organization has to make a decision whether they share data or not, the patient representatives are meaningfully involved in the decision-making process.

### 3.5 Data control

#### 3.5.1 Meaning of the notion “data control”

In total, 144 respondents provided answers to the open-ended question “What does control over the use of your personal data (also called data control) mean according to you?”. Four main concepts, often overlapping between each other, emerged. Participants understood data control as:

1. *Use and access*: control over the use (i.e., by whom, for what entities, at what time) and the access (by the individual whose personal data is processed for research or by other entities), including both the possibility to allow and to restrict such uses/access.
2. *Transparency*: being informed about how and by whom their data is used was seen as an important element of being in control.
3. *Consent*: for around one-fourth of the participants, control was understood as being able to consent to the use of their personal data and being able to withdraw consent.
4. *Privacy, confidentiality and data protection*: a portion of respondents associated data control with the responsibilities of the data controller to safeguard their fundamental rights to privacy and data protection. For instance, they reported that control is “protection of the data and insurance that it is only used for the intended purpose [research] it was collected for or agreed for” (MPO1), and “data controller safeguarding your data” (P5).

Additionally, other aspects were discussed. Firstly, for many, control was intertwined with the feeling of being *respected*. Secondly, participants felt a strong personal connection with their data, with some comparing it to being equal to donating bodily material. Thirdly, a few patients discussed control as the possibility of *opting-out* from research, or as data ownership (“I am the owner of my data”, P28). Finally, only one respondent brought up the possibility of *collective control*, referring to the control of “the ethics committee of the clinical trial site where the patient is treated and where he/she supplies his/her data” (C2).

#### 3.5.2 Data control tools

**FIGURE 7:**
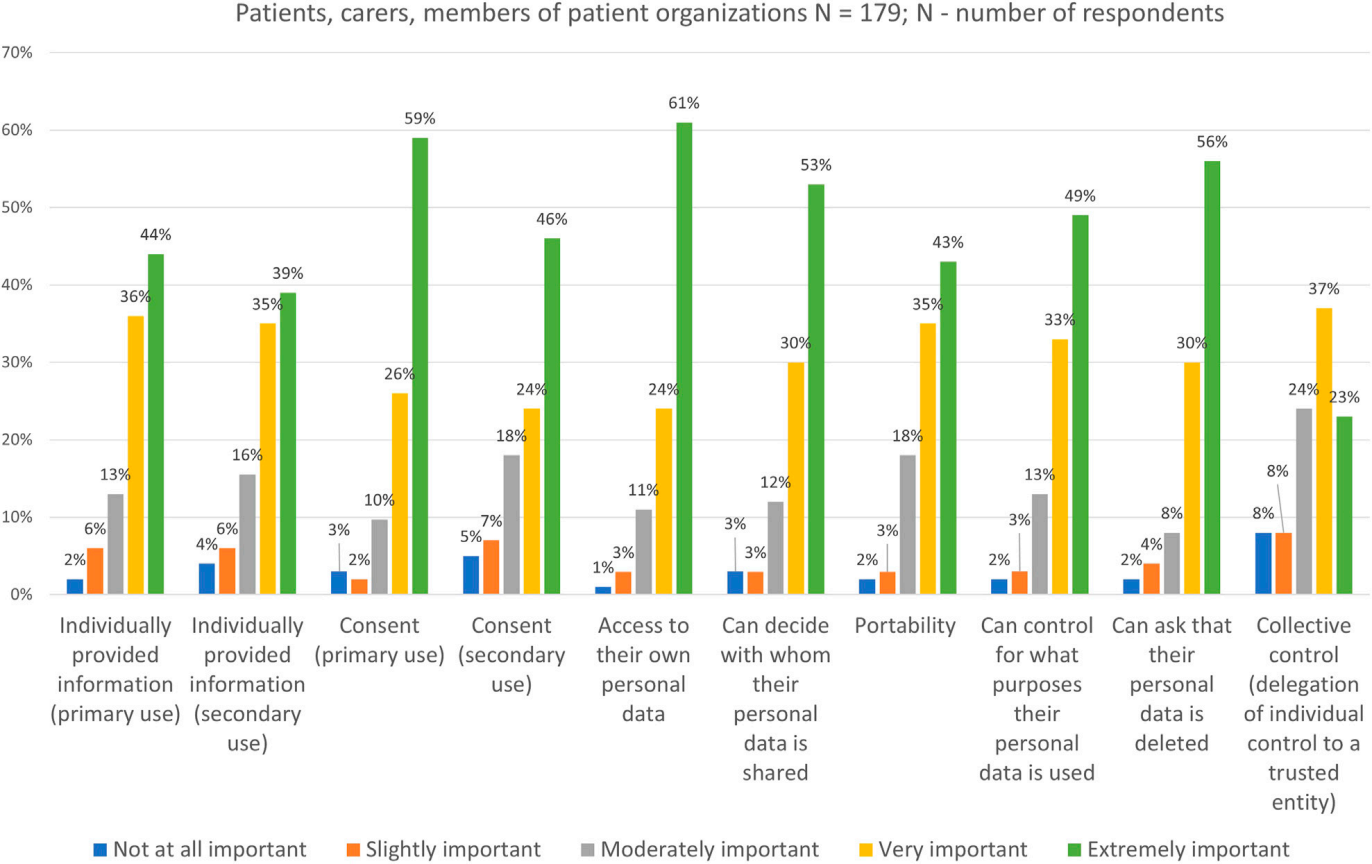
Participants’ views on the importance of different control tools.

Survey respondents were presented with a list of data control tools and asked to indicate on a 5-point scale how important each of the tools was to them in the context of medical research. The list included several individual tools (e.g., data subject rights such as information, access, or portability; consent as legal basis for the processing of personal data for primary or secondary use) and a collective tool (i.e., delegation of individual control to a trusted entity). Respondents highly valued all types of control tools (Figure 7). On average, almost half or more than half of the participants selected “extremely important” for each individual control tool, whereas only 23% (n = 41/179) thought that collective control is “extremely important”.

In addition, respondents could share which control tools, according to them, were missing from the list presented to them (open-ended question). A few participants added data subject rights provided by the GDPR, but not included specifically in the survey itself, particularly the right to withdraw consent and the right to rectification.

#### 3.5.3 Participants’ perceptions about the outcomes of individual data control

Participants were asked to share what positive and negative outcomes individual control could have for them (or the patient they take care of) and for health research in general. Several common themes were identified.

In terms of positive outcomes, respondents argued that individual control could lead to i) *benefits for individual patients* (e.g., better treatment decisions), as well as ii) *benefits for society* (e.g., improving clinical trials, fostering recruitment, increasing scientific knowledge). As stated by one patient, “more people might be willing to participate [in research] if they have control” (P68). Individual control was also linked to iii) *fostering trust* between patients and researchers, which was suggested to also lead to more patient participation in research overall and to boost the willingness to share data. Moreover, control leading to iv) *prevention of misuse of data and harm* (e.g., discrimination) was also often mentioned. Related to this theme, a few patients expressed that control would allow them to prevent the use of their data for research projects that they did not approve, even if this use was not per se harmful.

One of the most important concerns for patients was to be v) *engaged in research* (e.g., setting up research priorities), which individual control, in their view, would help them achieve. The right to data portability was used as an example of being engaged, i.e., exercising it “in case I want my data to be used in another research” (P98). Similarly to trust, engagement was seen as a way to foster the conduct of more research that aligns with the patients’ values.

Finally, respondents thought that individual control could contribute to vi) *ensuring the legally and ethically sound conduct of research*, particularly with respect to guaranteeing better protection of their fundamental rights to data protection and privacy. This outcome was discussed in a two-fold manner: on the one hand, control was linked to the responsibility of the individual patient to safeguard their own fundamental rights; on the other hand, control was viewed as a mechanism to drive research stakeholders towards respecting and addressing the individuals’ rights better.

With respect to negative outcomes, a key common thread in the participants’ answers was that there might be a risk for i) *jeopardizing the conduct of research*. For instance, fewer participants could be willing to participate in clinical trials, less data might be shared, and the use of some data subject rights (right to data portability or right to withdraw consent) might lead to biased data sets. Some participants expressed concerns about missed opportunities (both in terms of individual benefits and progress of research in general) by exercising control in a detrimental way due to lack of medical expertise.

Additionally, respondents saw as a negative outcome the ii) *burden control might present for individuals*, which could be either administrative (“There is so much data out there that (…) it would take way too much effort for me to track it all down”, P5), or psychological (“results or terminology not understood correctly by the patient might cause distress”, C1). Moreover, the possible iii) administrative *burden for research stakeholders* was also mentioned (higher workload and financial costs), specifically for public research institutions.

Finally, some participants were afraid of being iv) *punished for exercising control* (e.g., their access to tools or services being limited if they withhold consent), or v) of *their control being influenced* due to a power imbalance with other stakeholders: “Entities might try to buy me against my best interest or better judgment” (P15).

Overall, the risks associated with individual control, were summarized by one patient as: “If not managed properly, control over personal data may be seen as a hurdle by healthcare stakeholders, and perhaps contribute to the narrative that privacy and data protection is bad for innovation.” (P1).

## 4 Discussion

To our knowledge, this study is the first to investigate the awareness about the GDPR and the perceptions about data control among the patient community (patients, carers, and members of patient organizations who are neither patients nor carers) in the EU, EEA, and the United Kingdom. Responses were received from all four European regions (28 countries in total). However, most participants were based in Western and Northern Europe. Although there was a general high level of awareness about data protection, survey participants rarely used their data subject rights. The right of access and the right to data portability were the most commonly used among the survey sample. Respondents valued data control and showed a slight preference towards individual control tools. Strengthened patient involvement in the decision-making process of data intermediaries (in particular, data altruism organizations) appeared to be key for fostering the respondents’ willingness to share personal data. Finally, the empirical research results offered starting points for meaningful further reflection in the discussion not only on the current data protection legal framework, but also on specific provisions of the EHDS proposal.

### 4.1 High awareness about data protection

Awareness about the GDPR was generally high among respondents to the survey. This is in line with previous studies among the general population (e.g., in the Netherlands (Strycharz et al., 2020), Norway (Presthus and Sorum, 2021) and across Europe, as reported by Deloitte (Deloitte, 2018) and the Eurobarometer (European Commission, 2019)). The main sources of information about the GPDR were also aligned with evidence in the academic literature as being the news, cookie notifications, employers. Similarly to the conclusions made by Strycharz et al. (Strycharz et al., 2020), the results of this survey highlight the important role of media in advancing data protection and privacy literacy. However, unlike any other previous study, the most frequently cited source of information for our participants was specialized training. This could be explained by the demographic characteristics of the sample, i.e., most participants held a university degree, a significant portion of patients and carers were also members of patient organizations, and more than half of the participants had followed courses on the topics of medicines development, clinical research, or patient engagement. The results thus also signify that the importance of providing education about data protection and privacy is increasingly being recognized by patient organizations and advocacy groups. For instance, WECAN released a five-module training on the GDPR which focuses on, *inter alia*, what the GDPR is, why it matters, how data processing and protection work in practice.^22^ EUPATI also includes lessons on data protection in the course on “Legal, regulatory and health technology assessment concepts of digital health”.^23^

It is important to keep in mind that patients may have vastly different experience and technical knowledge about medical research, including data protection related issues. For these reasons, the EUPATI guidance for patient involvement in medicines research and development distinguishes between five groups of patients (Table 4): from individual patients (who have personal experience with living with the diseases) up to patient experts (who, in addition to disease-specific expertise, have knowledge in research and development). When it comes to individual patients who may not be members of patient organizations or who may not have followed specialized trainings, the role of other healthcare and research stakeholders in raising awareness about data protection must not be overlooked. In particular, healthcare professionals, nurses, and pharmacists – who are in closest contact with patients, as well as governmental bodies (given the increasing policy focus on facilitating and establishing trust in health data sharing, cf Introduction and Section 4.4).

**TABLE 4:** Definitions related to the term “patient”, as summarized by Warner et al. (2018).

Individual patients	Persons with personal experience of living with the diseases, who may or may not have technical knowledge about research and development (R&D) or regulatory processes
Carers	Persons supporting individual patients, e.g., family members as well as paid or volunteer helpers
Patient advocates	Persons who have the insight and experience in supporting a larger population of patients living with a specific disease and who may or may not be affiliated with an organization.
Patient organization representatives	Persons who are mandated to represent and express collective views of a patient organization on a specific issue or a disease area.
Patient experts	Persons who, in addition to disease-specific expertise, have the technical knowledge in R&D and/or regulatory affairs – for example EUPATI fellows, who have been trained on the full spectrum of medicines R&D.

Another unique source of information that was reported by more than one-fourth of participants was their experience taking part in medical research. This emphasizes the importance for research institutions to fulfil their transparency obligations under the GDPR. Our previous research has shown that research stakeholders find it difficult to be clear and concise when providing GDPR-related information for the primary use of personal data (Lalova-Spinks et al., 2022). It is important that further efforts are made towards optimizing the way data protection information is provided in the context of research. The use of legal design methods when drafting data protection and privacy notices for medical studies, and for the preparation of training materials for investigators, could be an appropriate way forward to achieve this (Rossi et al., 2019; Ducato et al., 2021; Lalova-Spinks et al., 2022). Legal design combines law, technology, and design to create user-friendly legal documents and, more broadly, to make the legal system more accessible to people (Rauccio, 2021).^24^

### 4.2 Rare use of data subject rights

Similarly to previous research among consumers (Deloitte, 2018; European Commission, 2019; Strycharz et al., 2020; Presthus and Sorum, 2021), our results showed a contrast between the high level of awareness about the GDPR, and the actual use and plans for future use of data subject rights by patients. There could be several explanations for this. For instance, Drechsler has outlined three main obstacles that impede data subject rights from fully reaching their potential as an empowering mechanism: 1) lack of awareness about the rights themselves, 2) lack of transparency about data processing, and 3) issues with enforcement, i.e., difficulties experienced by data protection authorities in handling data subject requests (Drechsler, 2023). Even if the overall awareness about the GDPR is high, more efforts are needed to help deepen the patient community’s specific knowledge about the intricate toolbox of data subject rights.

Furthermore, as discussed above, transparency in relation to data processing is already challenging for primary use, and this is exacerbated when personal data is reused for new projects and by complex chains of various controllers (Lalova-Spinks et al., 2022; Lalova-Spinks et al., 2023). A valuable best-practice example for ensuring better transparency could be found in the framework developed by the Belgian university hospitals for handling requests for secondary use of data (Raad van Universitaire Ziekenhuizen van Belgie, 2022). The framework is built upon six key conditions, and the right to information is one of them. In particular, it recommends that patients should be informed as much as possible about the secondary use of their data through a combination of different stages and levels of information. Such levels include general information provided in the hospitals’ privacy policy or information brochures and individual information about specific projects, ideally provided through digital solutions. In case of a prospective interventional experiment, the information about the data processing must be provided together with the informed consent for participation in the study (Raad van Universitaire Ziekenhuizen van Belgie, 2022).

Solove provides another explanation for the relatively low use of data subject rights in the general population, namely, that individuals do not care to exercise them (Solove, 2021). This aligns with the views of a limited number of investigators who suggested that patients are not interested in GDPR-related issues when joining a medical study, because their priority is to receive better care and treatment (Lalova-Spinks et al., 2022). A similar reason for not exercising data subject rights is echoed in this survey’s responses to the question exploring the negative outcomes of individual data control, namely, the administrative and psychological burden which control might present.

The possibility to exercise certain data subject rights can also be affected by the legal basis on which the processing of data occurred (European Organisation for Research and Treatment of Cancer, 2020; Lalova-Spinks et al., 2022), or by exemptions afforded by the GDPR and implemented in national law. For instance, for the right to data portability to apply, data must have been originally processed on the legal bases of consent or performance of a contract.^25^ Individuals whose data was processed based on a different legal ground would not be able to rely on their portability right. Another example pertains to the right to object. If the personal data was used for research purposes, the controller may refuse the request if the processing is considered to be necessary for public interest reasons.^26^

Finally, it should not be forgotten that the effectiveness of data subject rights does not depend on how many people exercise them per se (Strycharz et al., 2020). Many of the GDPR rights primarily serve to allow data subjects to hold processing entities accountable for their actions when they are not fulfilling their obligations in the first place (Gonzalez Fuster, 2015; Ausloos, 2020). On the other hand, while data subject rights are definitely a privacy preserving mechanism, they could also be seen as a mechanism to steer and impact medical research. For example, by exercising the right of access and the right to data portability to transmit data to other research institutes or projects than the ones in which they originally participated, individuals effectively exercise control over the type of research and research actors they support with their personal data. This directly links to the ongoing discussion about the new EU policy initiatives (such as the EHDS and DGA) introduced with the European Strategy for Data. The European Commission aims to facilitate access to and (re)use of data, including for medical research and for regulatory purposes (such as the assessment of medicinal products), while simultaneously empowering individuals, namely, by strengthening individuals’ control over their personal data (European Commission, 2020). It also appears that the Commission implicitly links the two objectives - i.e., by empowering individuals and increasing trust in the overall system, access to and (re)use of data will be enabled, and thus research fostered (Baloup et al., 2021). However, further interdisciplinary research is required to elucidate the relationship between data control (particularly understood as exercising data subject rights) and medical research in the EU.

### 4.3 Data control: a complex labyrinth

As mentioned in the Introduction, even though the notion of control is central to the GDPR, it presently lacks a clear definition. Respondents discussed control through four main concepts: 1) use and access, 2) transparency, 3) consent, and 4) privacy, confidentiality, and data protection.

These concepts broadly align with the control model discussed by Vayena and Blassime (Vayena and Blasimme, 2017). The authors proposed a working definition of control: “the power to decide on the conditions of exposure of health data and personal health information”. They unpacked the notion of control over three dimensions: 1) control over access (who gets access), which could be exercised, e.g., through the right to data portability, 2) control over data use (what is data used for), which could be exercised through, e.g., electronic informed consent and dynamic consent models^27^, and 3) control through participatory governance schemes, such as collective control. According to Vayena and Blassime, while each of these dimensions is promising, it is not a sufficient condition for control in itself – rather, they should work together.

Our findings also generally correspond with the results of the recent citizen consultation conducted within the scope of the TEHDAS’ “Healthy data” project. The project aimed to collect citizens’ and patients’ views on the secondary use and sharing of health data, and on the role that they would like to play in the management and use of their health data (Menager et al., 2023). It was open for contributions from all EU Member States, but input primarily came from individuals residing in Belgium, France, and the United Kingdom. The outcome consisted of a set of twelve recommendations on how to engage citizens in the European Health Data Space (see summary in Table 5). The recommendations are clustered around three main concepts: 1) the data relationship, 2) power balance, and 3) a citizen-powered framework. The notion of control is found in Recommendation 6: “Citizens should be provided with the opportunity for meaningful and active decision-making in the secondary use of health data”. Our results are in support of this recommendation. Namely, as shown in the example concerning data altruism, the involvement of patient representatives in data altruism organizations largely increased the willingness of respondents to share data.

**TABLE 5:** Recommendations on how to engage citizens in the European Health Data Space (summary of Menager et al., 2023).

The Data Relationship
1. Citizens should be able to access information about the secondary use of health data, in an understandable way, allowing them to be more engaged.
2. Citizens should have access to their data and know how they are used for secondary purposes.
3. Citizens’ values should inform what is beneficial to individuals and what constitutes the common good.
4. Decision-making processes should rely on a plurality of views and actors to increase their trustworthiness.
5. Citizens should be given the opportunity to be involved in the lifecycle of health data.

The Power Balance
6. Citizens should be provided with the opportunity for meaningful and active decision-making in the secondary use of health data, as they value their ability to exercise control.
7. The protection of individuals’ identity should be ensured; citizens perceive this as one of the best ways to balance the harms and benefits of the secondary use of health data.
8. Data users’ intentions should be transparent and in line with purposes of citizens’ support.
9. Accountability could be enhanced through transparent and stronger mechanisms.
10. Good IT solutions should be fostered to protect citizens’ data, beyond having a strong legal framework in place.

A Citizen-Powered Framework
11. Stakeholders should respect principles that align with citizens’ ethical values.
12. There should be a framework that facilitates the secondary use of health data for purposes and benefits that citizens support, while minimizing the potential risks that citizens’ identify.

However, it is interesting to observe that most of the actions and terms that our survey respondents associated with data control or with the outcomes of control appear scattered across the three TEHDAS clusters. For example, access, transparency or engagement in research would fall under the Data Relationship, whilst fostering research that aligns with patients’ values could fall under the Citizen-Powered Framework cluster. This discrepancy illustrates that, while the key forming elements of control are identifiable through empirical and doctrinal research, more fundamental work is required to determine the precise conceptual underpinnings and clearly construct the theoretical notion of data control (Ducuing et al., 2021).

The body of academic literature on patients’ attitudes towards data sharing (Kalkman et al., 2022) can also be an important element in the discussion about control. The survey participants’ perceptions about the positive and negative outcomes of control strongly align with empirically elucidated reasons, motivations, and perceived risks for health research data sharing. For instance, numerous past studies have shown a wide range of different motivations for data sharing, such as contributing to advancements in healthcare and scientific knowledge, the hope for future personal health benefits and the wish to assist the common good (Haga and O'Daniel, 2011; Shabani et al., 2014; Goodman et al., 2017; Mazor et al., 2017; Mursaleen et al., 2017; Stockdale et al., 2018; O'Brien et al., 2019; Richter et al., 2019). This is in line with the positive outcomes of data control identified in terms of individual and societal benefits by the responses to our survey. Another example pertains to the perceived risks of data sharing, mainly focused on fear of breaches of confidentiality, commercial use of data, and potential misuse of the data (Haga and O’Daniel, 2011; Joly et al., 2015; Aitken et al., 2016; Spencer et al., 2016; Sanderson et al., 2017; Goytia et al., 2018; Howe et al., 2018; Colombo et al., 2019). These all correspond to the negative outcomes of data control shared by our survey participants. The illustrated relationship could be an indication of a perceived connection between control and data sharing, i.e., that more control could promote more data sharing (the same argument is put forward by, e.g., Vayena and Blassime (Vayena and Blasimme, 2017)). This would be in line with the European Commission’s implicit goal, described above: enabling data for reuse through the empowerment of individuals. Finally, the insights about factors that affect data sharing willingness are a crucial feature of studies about data sharing attitudes. These factors include, e.g., age, geographical factors, socio-economic background, etc (Kalkman et al., 2022). It was not in the scope of this study to elucidate such factors in relation to data control perceptions, but they could constitute an important future research line.

Survey respondents highly valued both individual and collective data control tools, with a slight preference for individual tools. However, as identified by our participants and confirmed in literature, there are risks to individual control, particularly when control is framed as either a personal responsibility which might result in an excessive administrative and psychological burden for individuals (Rauhofer, 2008; Rouvroy et al., 2009; Brunton and Nissenbaum, 2016; Vayena and Blasimme, 2017) or when it is merely illusory, e.g., control through consent that has not been obtained validly, for instance in situations when genuine choice is missing or where there is an imbalance of power between the participant whose data is being used and the person/entity undertaking the research (Brandimarte et al., 2009; Solove, 2012; Verhenneman, 2021). In addition, it must be borne in mind that the fundamental right to data protection is not absolute – the law balances the rights and interests of individuals with respect to the use of their personal data and the rights of others (e.g., use of data for research). Due to this fact, “good mechanisms must be put in place to ensure that individual rights are not sacrificed at the altar of alleged public interest”, as put by Prainsack et al. (Prainsack et al., 2022a).

#### 4.3.1 Data solidarity: a potential solution of the individual vs collective control conundrum

To overcome the limits of individual control tools, scholars and practitioners are increasingly discussing the importance of strengthened collective empowerment, such as ceding control to another party (Vayena and Blasimme, 2017; Mahieu and Ausloos, 2020; Kickbusch et al., 2021; Rerolle and Roussoulieres, 2022; Solove, 2023). More work is necessary to critically evaluate existing collective control tools, and to further promote awareness about them among the patient community. One of the mechanisms by which collective control could be meaningfully discussed in the future, is data solidarity. In the case of medical research, scholars consider that data solidarity should be taken as a blueprint for data governance (Prainsack et al., 2022b). Solidarity is defined as a practice that comprises people’s commitments to supporting others with whom they recognize similarity in a relevant aspect (Prainsack and Buyx, 2011; Prainsack and Buyx, 2017). Solidarity-based data governance is one which ensures that the benefits and costs of data use are borne collectively and fairly (Prainsack et al., 2022b). Data solidarity’s focal points are harm prevention and the creation of public value by specific instances of data use. Public value is created when the use of data benefits people without posing heavy risks. Under the framework proposed by Prainsack et al., data uses that are likely to benefit the public considerably, and which pose no heavy risk, should be supported, whereas data uses that pose grave risks while creating little or no public value, should be prohibited. Data solidarity is proposed as a guiding principle to get out of the dichotomy between individual interest and societal good, which is thought to be unproductive. It posits that the relationship between self-interest and concerns directed towards others is complementary rather than a tradeoff (Prainsack et al., 2022b).

#### 4.3.2 Learning from patient empowerment in the broad sense

Finally, when it comes to enabling meaningful (individual and collective) control, some lessons can be learned from the debates surrounding patient empowerment in a broader sense. For patient empowerment to be successfully operationalized, several enablers are necessary, such as achieving a common understanding of the term itself, enhancing health literacy and education, effective communication about empowerment, addressing cultural barriers, communicating about the individual benefits for each patient, and providing structural support and resources (Hoos et al., 2015; Geissler et al., 2017; Haerry et al., 2021). By analogy and based on the findings of this study, data control requires the same prerequisites for its meaningful practice.

### 4.4 A look into the future: the European Health Data Space

As established earlier, increasing (individual) control is one of the key objectives of the proposed EHDS.^28^ Thus, it is pertinent to briefly discuss the draft regulation in view of the results of this study.

#### 4.4.1 Enhanced individual data control … , or not?

The EHDS rules pertaining to the primary use of data (i.e., for healthcare or social security purposes) clearly contribute to strengthened patient empowerment (Lalova-Spinks, 2023). Chapter II of the EHDS proposal focuses on rights and mechanisms that complement existing data subject rights. These mechanisms will enable natural persons to obtain better care by reducing information asymmetries between providers and users of health services, and by facilitating informed choice (Marcus et al., 2022). An important example pertains to data portability, which will be significantly enhanced. Namely, it will be possible to exercise the right irrespective of the legal bases on which the original data processing took place, as well as to transmit not only individually provided data, but also inferred and derived data (e.g., medical examinations).^29^ Enhancing the portability of data stored in electronic health records will presumably contribute to, e.g., patients obtaining more meaningful specialist opinions or second opinions on their disease and treatment (Marcus et al., 2022). Such a consequence is in line with one of the key positive outcomes of individual data control identified in the survey, i.e., “benefits for individual patients”. However, there is no explicit indication whether the broadened right to data portability, along with all other mechanisms that complement data subject rights, could be exercised in the context of secondary use of data (Chapter IV), and thus also for medical research.

The supporting documents accompanying the draft EHDS proposal do not provide information about why the legislator opted not to broaden data portability for secondary use. The focus on primary use only seems to go both against the overarching objective of the EHDS for increasing individual control, irrespective of the situation, and against the European Strategy for Data’s aim to provide every citizen with portability of their data (European Commission, 2020). On the one hand, a possible explanation could lie in one of the fears shared by our survey respondents, namely, that individual control could be negatively influenced due to power imbalances with other stakeholders. Individuals could be prompted to transmit their health data against their best interest, for example. The new law could address this risk while maintaining a balance between empowerment and protection of patients through, e.g., offering a broadened right to data portability for secondary use but limiting the purposes for which it can be used. Such purposes could be, for instance, scientific research conducted in the public interest.

On the other hand, the explanation could be linked to preserving clinical research validity and integrity. For instance, if participants in a clinical trial transfer their data from the trial sponsor to another entity, it would not be clear whether their data can still be included in the final analysis, whether the results of the interim analysis can be re-done to verify correctness, or whether the data can be used in the scope of an inspection by competent authorities (European Organisation for Research and Treatment of Cancer, 2020; Lalova-Spinks et al., 2023). In addition, premature clinical trial data leaking could lead to biased assessment of treatment outcomes. While it is of utmost importance that clinical trial data is shared, sharing must only occur after appropriate validation and after analysis of the primary endpoints, in order not to jeopardize the validity of the trial itself. However, if indeed preserving clinical trials validity and integrity was the underlying policy choice behind limiting the right to data portability in all scientific research cases, this must be made explicit. Moreover, the right could still be strengthened for scientific research, while introducing a limitation specifically for the case of clinical trials. The GDPR itself already took into account the need for a balanced use of the portability right, as pursuant to Article 20(4), data portability shall not adversely affect the rights and freedoms of others.

#### 4.4.2 Addressing transparency

Although transparency is key for putting individuals in control of their personal data (as shown throughout the results and discussion), the EHDS provides health data access bodies with a broad exemption from the obligation to provide each natural person with information about the reuse of their data.^30^ Only general public information about all issued data permits will be required. As discussed by Slokenberga, this exemption risks undermining data subject rights (Slokenberga, 2022). The European Data Protection Board and the European Data Protection Supervisor already criticized this provision and called on the legislator to specify the concrete conditions under which the exemption would be available (EDPB-EDPS Joint Opinion, 2022). The amendments to the EHDS text proposed in the draft report by the ENVI and LIBE committees’ rapporteurs at the European Parliament go in the direction of tipping the balance towards strengthened individual control. Instead of the blanket exemption for health data access bodies, the draft report suggests that an exception applies only if “the provision of information (…) to each natural person concerned proves impossible or would involve a disproportionate effort in accordance with Article 14(5)(b) [GDPR]”.^31^

#### 4.4.3 Consent vs. opt-out

Around one-fourth of our survey respondents associated data control with providing consent for the processing of their data, and a few – with the possibility to opt-out from research. At present, consent and opt-out are hotly debated as two of the possible models for legitimizing the secondary use of personal data under the EHDS (Kogut-Czarkowska and Fiers, 2023), whilst safeguarding the individual data control of patients. However, neither was part of the first draft of the proposed regulation.

The EHDS aims to provide the legal basis for the secondary use of data,^32^ whilst deliberately moving away from consent.^33^ It seems that the proposal intends to supersede any national provisions that explicitly require consent. Namely, pursuant to Article 33(5) of the EHDS, “Where the consent of the natural person is required by national law, *health data access bodies shall rely on the obligations laid down in this Chapter [IV] to provide access* to electronic health data.” [authors’ emphasis]. A few remarks are merited here. First, in our view, it is unclear whether the cited provision refers to consent as a legal basis under the GDPR, research ethics consent, or even both types of consent. A clearer phrasing of the pertinent article(s) would be necessary to achieve legal certainty in an already highly complex field. Second, when it comes to GDPR consent, it can only be considered as the suitable legal basis if it fulfils the requirements for valid consent established in the law, i.e., freely given, specific, informed, and unambiguous.^34^ Moreover, consent under the GDPR does not hold a privileged position, but is one among six legal grounds that can legitimize the processing of personal data.^35^ As pointed out by Verhenneman, the situations in which consent can be considered a valid and preferred legal basis are rare, e.g., it would always fail in a situation of power imbalance (Verhenneman, 2021). Patient empowerment is not achieved exclusively through consent and other means might play a more important role, such as the principle of purpose limitation (Verhenneman, 2021).

One way forward, suggested in the draft ENVI and LIBE committees report, is to introduce an opt-out mechanism in the EHDS, “whereby natural persons must be offered the possibility to explicitly express their wish not to have all or part of their personal electronic health data processed for some or all secondary use purposes”.^31^ In their justification of the proposed amendment, the committees focused precisely on preserving the essence of the right to data protection and providing for suitable and specific measures to safeguard the fundamental rights and interests of data subjects, as well as on safeguarding the trust between patients and their healthcare providers (as “patients may no longer wish to share their health data with healthcare providers if the data are then automatically passed on for secondary use”).^31^

Historically, opt-out is a mechanism known in the biobanking field, with practical roots. Current biomedical research calls for the establishment of large pools of tissue and samples; if the human body material stored by biobanks is unrepresentative of society as a whole, future treatments for those not represented are likely to become increasingly scarce (Kozlakidis et al., 2012). Whilst according to some, opt-out is a practical way to address under-representation (Johnsson et al., 2008; Pulley et al., 2010; Kozlakidis et al., 2012), others warn against precisely the opposite, i.e., that opt-out models could in fact have a negative effect on participation rates due to, e.g., public distrust (Beyleveld and Buchanan, 2007). At present, both opt-out and consent models are practiced in the biobanking field (van Veen et al., 2006). An example for an opt-out model established into national law can be found Belgium, where research ethics consent is presumed for residual use of samples,^36^ unless the donor has announced his or her refusal prior to any operation involving the material.^37^ A sensibilization campaign will aim at informing the general public about the nature and aims of biobank research, similar to organ donation campaigns in the past (Lalova et al., 2021).

The revised CIOMS guidelines recognized the possibility to rely on an informed opt-out procedure not only for the use of biological material,^38^ but also for the reuse of data collected in the context of routine clinical care.^39^ The guidelines identify four conditions that must be fulfilled for valid opt-out namely: 1) patients need to be aware of the existence of opt-out; 2) sufficient information needs to be provided; 3) patients need to be informed that they can withdraw their data; and 4) a genuine possibility to object has to be offered. However, the guidelines also recognize that, due to the diversity of research situations, not all cases can be treated alike, and in certain circumstances researchers are expected to obtain explicit informed consent, in particular 1) when the research involves more than minimal risks to the individual, 2) when controversial or high-impact techniques are used, or 3) when research is conducted in the context of heightened vulnerability.^40^

With respect to the EHDS, if the legislator chooses opt-out as the preferred model for secondary use, the conditions identified by CIOMS might serve as a valuable basis for building up the mechanism. However, the practical implementation of working mechanisms, based on the aforementioned conditions, might be a challenging task for the policymakers. For example, large investments and considerable efforts would be necessary to build the required infrastructure for operationalizing opt-out, to ensure sufficient transparency and to launch EU-wide sensibilization campaigns.^41^ Furthermore, as increasing the participation in research through an opt-out model is contingent on public trust (Beyleveld and Buchanan, 2007), community engagement would be paramount for the successful implementation of opt-out.^42^ However, the EHDS in its current draft is not strong on its stakeholder involvement provisions (see below “Collective data control in the EHDS”).

It is important to acknowledge that both opt-out and consent bear the risk of limiting the representativeness of datasets in the EHDS from its inception, consequently potentially compromising study outcomes. (Marelli et al., 2023). As mentioned above, the first draft of the EHDS proposal severely undermines consent and does not discuss opt-out. Whilst not relying on either model might at first glance appear against the objective of strengthened individual control, this would not necessarily be the case as long as another valid legal basis for the processing of personal data can be identified under the GDPR, the interplay of the EHDS with the GDPR is clear enough to not jeopardize the application of the key data protection principles, and an adequate governance framework and appropriate safeguards are put in place.

#### 4.4.4 Collective data control in the EHDS

Finally, the EHDS proposal allows to pay attention to broader forms of patient empowerment, i.e., collective control. Stakeholder involvement is one of the possible ways to establish trust in data sharing and the data governance system (Lalova-Spinks et al., 2023). Not surprisingly, our survey participants indicated that if patient representatives were involved in the decision-making process of data altruism organizations, they would be likely to share personal health data with such organizations. Although the adopted DGA fails to clearly mandate the involvement of citizen and patient representatives in data altruism organizations, the European Commission has recognized the value of such engagement when it comes to health data access bodies in the proposed EHDS. Pursuant to Article 36(3), health data access bodies shall “actively cooperate with stakeholders’ representatives, *especially with representatives of patients*” [authors’ emphasis]. However, this formulation of the obligation lacks further specification, which might jeopardize its application in practice. The amendments proposed by the ENVI and LIBE committees mitigate the weak phrasing by transforming the provision into: “Member States shall ensure that essential health stakeholders’ representatives, including patient organizations and healthcare professionals shall be present in the governance and decision-making structures of the health data access bodies (…)”.^31^ As the EHDS will be the law to provide specific rules for the application of data altruism in health, in our view, the legislator should use the opportunity to extend the obligation for patient involvement also to data altruism organizations.

#### 4.4.5 The way forward for the EHDS?

Ultimately, it is not surprising to encounter certain limitations on data subject rights when it comes to secondary use of data under the EHDS, due to the non-absolute nature of the fundamental right to data protection. However, such limitations should occur when they are well justified, necessary, and proportionate. In addition, the almost complete lack of provisions that strengthen patient empowerment, and individual data control in the first draft of the proposal goes against the objectives of the EHDS proposal itself. As Slokenberga suggested, either the mechanisms outlined in Chapter IV must be revised to grant individuals some control, or the scope of Article 1(2)(a) should be explicitly limited to the primary use of electronic health data (Slokenberga, 2022). The EHDS proposal is an ambitious piece of legislation that promises to bring new opportunities for healthcare, research, and patient empowerment. Currently, it is undergoing substantial revisions and thus it remains to be seen how the final version will strike a balance between increased individual data control and (re)use of personal data in the context of research. Approaching the construction of the new legal framework through data solidarity lenses could be a valuable way forward. In particular, building a well-defined governance framework that should be able to ensure reuse of data that is highly likely to create public value without posing high risks to individuals or groups (Marelli et al., 2023).

## 5 Future research

The discussion of our survey results outlined several avenues for future research. Further qualitative research (such as semi-structured interviews or focus group discussions with representatives of the patient community) and mixed methods research is needed to better understand, e.g., the reasons behind the low rate of exercising data subject rights, or to study the approval of citizens as regards opt-out for secondary use of personal data in the EHDS proposal. Moreover, studies into the factors (such as age, socio-economic background, etc.) that impact data control perceptions and willingness to exercise different types of control are necessary. Additionally, legal doctrinal research into the notion of data control and its operationalization in current and proposed legislative initiatives is highly important (Ducuing et al., 2021).

It is crucial to continue elucidating the knowledge and perspectives of patients, carers, and members of patient organizations about data protection on a larger scale, i.e., by focusing on underrepresented regions such as Southern and Eastern Europe, and by involving patients with different backgrounds, levels of education, and personal experiences. The inclusion of more diverse patient voices can be facilitated by building long term partnerships with communities through different levels of outreach and engagement, including participatory science, co-design and co-production. For example, ensuring that questionnaires are available in the different languages of the EU Members States as well as through both offline and online channels, would be an asset.

Finally, the rise of AI applications in healthcare and research necessitates further investigation on how such AI tools might, on the one hand, be used in improving care based on individual data points, and, on the other hand, how they might contribute to better data control processes, while also taking into account the risk such models – particularly “black box” ones – may pose.

## 6 Strengths and limitations

This study fills an important gap in current data protection and patient empowerment scholarship, as it provides a first look into the awareness about the GDPR and perceptions about data control among the European patient community. The study aimed to be inclusive and gathered input not only from patients, but also from patient carers and members of patient organizations, thereby taking into account that the patient journey includes their family and broader support network. Moreover, the survey was designed by a multi-disciplinary team which included patient experts, thereby aiming to respect the vulnerabilities and sensitivities of the studied population.

Nevertheless, the study also has some limitations. First, it was not possible to cover all targeted countries equally, which could be due to the dissemination strategy. Moreover, the questionnaire was made available only in English. Therefore, important perspectives might have been missed. The sample included predominantly highly educated participants, and thus their answers might not reflect the majority of the patient population which may be not be as aware of the data protection framework. Additionally, the discussion provided a critical outlook at a proposed piece of legislation, the EHDS, that is not yet final. The proposed regulation might still change; however, it was deemed important to already put it to the test in view of the survey results and the ongoing legal debates.

## 7 Conclusion

The article examined European patients’ awareness about and perspectives on data protection and data control. While the awareness about the GDPR itself was high, data subject rights were rarely exercised. The results highlighted the importance of providing in-depth education about data protection and privacy, a crucial role for which could be played by patient organizations and advocacy groups.

Participants valued data control and identified positive and negative outcomes of its use. Positive outcomes included, e.g., benefits for individual patients (e.g., better treatment decisions), benefits for society (e.g., fostering recruitment in clinical trials and increasing scientific knowledge), fostering trust in research, and prevention of misuse of personal data. As potential negative outcomes were mentioned, e.g., the administrative or psychological burden data control might present to individuals, the administrative burden for researchers, and the risk of control being influenced due to power imbalances in the relationship of patients with other stakeholders.

Although respondents showed a slight preference towards individual data control tools, the reflection based on existing research identified that individual control holds risks that could be mitigated through carefully operationalized collective tools. In relation to this, the increased patients’ willingness to share personal data with data altruism organizations if patient representatives would be involved in the decision-making processes of such organizations was an important finding.

Furthermore, effective data control could be seen to rely on enablers similar to the ones that foster meaningful patient empowerment in a broader sense. These include, but are not limited to, education, effective communication and addressing cultural barriers.

Finally, the results from this work provided a starting point for a critical discussion of the proposal for a European Health Data Space regulation, which has yet to find a productive balance between individual control and facilitating the secondary use of data.

## Data Availability

The datasets presented in this article are not readily available because of legal and ethical constraints. The survey data can be made available to other researchers only upon request, ensuring that the information is not used for secondary data analysis without the prior consent of the research team who conducted this original study. Requests to access the datasets should be directed to teodora.spinks@kuleuven.be.

## Footnotes

1 Clinical trials are strictly regulated and subject to mandatory ethical oversight. In this regard, the international ethics and human rights framework is key. Additionally, several layers of legislative acts interplay. First, the regulations and directives adopted at EU level, e.g., the Clinical Trials Regulation 536/2014, the Medical Device Regulation 2017/746, the In Vitro Medical Devices Regulation 2017/746, and the General Data Protection Regulation. Furthermore, the EU legislator has been active in the field of scientific research and data governance. New policy and legal initiatives, as first outlined in the European Strategy for Data, are coming up, such as the European Health Data Space proposal or the recently adopted Data Governance Act (Regulation 2022/868) and AI Act. Finally, each of the 27 EU Members States have national laws in the field of scientific research that are often conflicting. For instance, biobanking is crucial for clinical trials but legislation for it is not harmonized at EU level.

2 For an in-depth discussion, see also Slokenberga S. Setting the Foundations:Individual Rights, Public Interest, Scientific Research and Biobanking in Santa Slokenberga, Olga Tzortzatou and Jane Reichel (eds), GDPR and Biobanking: Individual Rights, Public Interest and Research Regulation across Europe (Springer International Publishing 2021).

3 Regulation (EU) 2022/868 of the European Parliament and of the Council of 30 May 2022 on European Data Governance and amending Regulation (EU) 2018/1724 (Data Governance Act).

4 Article 1, Recital 3 and 5 DGA.

5 Article 2(8) DGA.

6 Article 2(16) DGA.

7 Recital 50 DGA.

8 Proposal for a Regulation of the European Parliament and of the Council on the European Health Data Space COM/2022/197 Final (2022).

9 Chapter II EHDS proposal.

10 Chapter IV EHDS proposal.

11 Guideline 7 CIOMS Guidelines.

12 Recital 7 GDPR.

13 Article 6(1)(a) and Article 9(2)(a) GDPR.

14 Chapter III GDPR.

15 Article 13 and 14 GDPR.

16 Recital 30 DGA, Article 10(b) DGA.

17 Recital 31, Article 10(c) DGA.

18 This questionnaire was part of a larger study, also incorporating questions on the topic of real-world data (RWD) and real-world evidence (RWE). The background and results with respect to RWD and RWE will be described in a separate publication.

19 Both questions allowed the selection of multiple answers. Due to this, note that in the results, the percentages for this type of questions do not add up to 100%, as the categories were not mutually exclusive.

20
https://connect.eupati.eu/

21 Display logic allows to set the condition that must be met for a survey question to be displayed. For instance, the condition could be based on an answer to a previous question. In our survey, for example, only respondents who answered “Yes” to the question “Have you ever heard of the EU General Data Protection Regulation (GDPR)?”, were presented with the follow-up question “What is your main source of information about the GDPR?”.

22 More information available at: https://academy.wecanadvocate.eu/.

23 More information available at: https://learning.eupati.eu/course/view.php?id=66.

24 For examples of legal design tools and projects, see the work of the Stanford Legal Design Lab: https://www.legaltechdesign.com/.

25 Article 20 GDPR.

26 Article 21(6) GDPR.

27 Valuable lessons about the employment of dynamic consent as an empowering tool with respect to data processing can be drawn from the use of dynamic consent for participation in biomedical research and biobanking. Past studies have shown that dynamic consent offers opportunities for ongoing communication between research participants and researchers, and that it supports cross-border research and large-scale data sharing. See, e.g., Budin-Ljøsne, I., Teare, H.J.A., Kaye, J. et al. Dynamic Consent: a potential solution to some of the challenges of modern biomedical research. BMC Med Ethics 18, 4 (2017); Biasiotto R, Pramstaller PP, Mascalzoni M. The Dynamic Consent of the Cooperative Health Research in South Tyrol (CHRIS) Study: Broad Aim Within Specific Oversight and Communication. BioLaw Journal - Rivista Di BioDiritto, n. 1S:277–87 (2021).

28 Article 1(2)(a) EHDS proposal.

29 Recitals 5, 12 and Article 3(8) EHDS proposal.

30 Article 38(2) EHDS proposal.

31 European Parliament, Committee on the Environment, Public Health and Food Safety & Committee on Civil Liberties, Justice and Home Affairs, Draft report on the proposal for a regulation of the European Parliament and of the Council on the European Health Data Space, (COM(2022)0197 – C9-0167/2022 – 2022/0140(COD), 10 February 2023, available at: https://www.europarl.europa.eu/doceo/document/CJ43-PR-742387_EN.pdf.

32 For data holders (e.g., sponsors of clinical trials, or hospitals), the EHDS constitutes the legal obligation for the valid sharing of data with health data access bodies, and moreover, the regulation intends to meet the conditions for processing special categories of personal data under Article 9(2) (g),(h),(i)(j) GDPR. The individuals or entities who wish to reuse data (i.e., data users) should demonstrate a legal basis under Article 6(1) GDPR: either (e)–public interest, or (f)–legitimate interests of the controller. In case the data user relies on Article 6(1)(e), they should make a reference to another EU or national law. However, if they rely on Article 6(1)(f), the EHDS will provide the necessary safeguards. In all cases of data users’ access to data, the EHDS would again provide the necessary condition under Article 9(2) GDPR. For health data access bodies the recognized legal basis for sharing data will be Article 6(1)(e) (public interest) GDPR, in combination with Article 9(2) (h),(i)(j) of the GDPR. See Recital 37 EHDS proposal.

33 See, e.g., the EHDS explanatory memorandum: “On secondary use of electronic health data, researchers, innovators, policymakers and regulators would be able to have access to quality data for their work in a secure way, with a trusted governance and at lower costs *than relying on consent*.” [authors’ emphasis].

34 Article 7 GDPR.

35 Article 6(1) GDPR. For the processing of special categories of personal data, such as data concerning health, a condition under Article 9(2) GDPR must also be identified.

36 Residual use means the use for research purposes of material that was collected for the purposes of diagnosis or treatment and would otherwise be destroyed. Article 2(33) of the Belgian Law regarding the procurement and use of human body material of 19 December 2008/*Loi relative à l’obtention et à l’utilisation de matériel corporel humain destiné à des applications médicales humaines ou à des fins de recherche scientifique*, hearafter Belgian Biobank law.

37 Article 20(2)(1) Belgian Biobank law.

38 Guideline 11 CIOMS Guidelines.

39 Guidelines 12 CIOMS Guidelines.

40 Commentary on Guideline 12 in CIOMS Guidelines.

41 Joint statement of 32 European patient organizations, medical associations, research organizations, data collaborations and industry associations: enabling effective secondary use of health data in Europe: specific recommendations for a potential opt-out mechanism for the EHDS. Available at: https://cdn.digitaleurope.org/uploads/2023/06/EHDS-statement-multistakeholder-group-6-June-2023.pdf.

42 Guidelines 7 CIOMS.
