# Supplementary material for: Patients’ knowledge, preferences, and perspectives about data protection and data control: an exploratory survey
Source: Front Pharmacol. 2024 Feb 20;14:1280173. doi: 10.3389/fphar.2023.1280173 (PMC10912650; doi:10.3389/fphar.2023.1280173)
Supplement: Supplementary file 1 [file DataSheet1.DOCX]

**Supplementary material 1**

**Survey questionnaire**

**Investigating patients' knowledge and perspectives about data control, real-world data and real-world evidence**

 **Welcome to our survey!**

 This survey is conducted in the context of research at KU Leuven, a university located in Belgium.

 **The purpose of this survey** is to gain insights into:
  
1) the knowledge and perspectives of patients, carers and patient representatives about the General Data Protection Regulation and about control over personal data in medical research, and

 2) their perceptions of real-world data and real-world evidence.
 **Patients, carers and patient representatives are important stakeholders in the field of medical research**. However, their views on the topics of data control, real-world data and real-world evidence are not sufficiently captured and described in existing academic literature. This survey will allow us to fill this gap and to make patient-centred policy recommendations relating to these topics. **The results of this survey will be used** **solely for scientific purposes**. They will be published in research articles and in the PhD dissertations of the researchers involved, and presented at academic conferences.

 The survey will take you **approximately 25 minutes** to complete.
 If you are unable to complete the survey in one session, you can click on the link later and you will be able to continue the survey on the page where you left off.

 Participation in the survey is **voluntary**. You can stop at any time, without any consequences for you or your care.

 The survey is aimed at individuals currently living in a Member State of the**European Union,** or in **the United Kingdom,** **Switzerland, Norway, Iceland or Liechtenstein**. Participants should be **at least 18 years old**. 

 The survey will **not collect any directly identifying information** about you unless you voluntarily provide such information. 

 The study was approved by the Ethics Committee Research UZ/KU Leuven (S66701).
Ethics committees verify if the rights of participants are respected by researchers during a study, if the balance between risks and benefits is beneficial for the participants and if the study is scientifically and ethically justified.

 In the scope of this survey, KU Leuven will process your data as data controller for the purposes that were presented to you. The legal basis for processing your personal data is public interest (Article 6(1)(f) of Regulation (EU) 2016/679).

 All research data will be stored for a period of 10 years. Your personal data may be stored for an additional period of time, if necessary, for the purposes of this study, or for further research on the topic.

 For more information, please refer to [KU Leuven privacy policy](https://admin.kuleuven.be/privacy/en) and [Qualtrics privacy policy](https://www.qualtrics.com/support/survey-platform/getting-started/data-protection-privacy/).

 If you have any further questions regarding this study, please do not hesitate to **contact**:
***Teodora Lalova-Spinks***, PhD researcher KU Leuven (teodora.lalova@kuleuven.be or +32 16 37 46 69)
 ***Robbe Saesen***, PhD researcher KU Leuven (robbe.saesen@kuleuven.be or +32 27 74 15 48)

 The survey is conducted in the scope of the PhD project of Teodora Lalova-Spinks, **funded with a PhD scholarship by the Research Foundation - Flanders (FWO)**, project number 11H3720N. 
 Thank you for participating in this project!
  
 **Do you agree with the terms presented to you above? You can only take the survey if you click "Yes".**

- Yes (this option allows you to proceed with the survey)
- No (this option automatically terminates the survey)

**End of Block: Introduction**

**Start of Block: Introductory questions to select target participants**

**Introductory questions**

Which of the following characterises you best?
 *(If you are both a patient and a carer, please choose from which perspective you would like to complete the survey)*

- I am a patient
- I am a carer
- I am neither a patient nor a carer

*Display This Question:*

*If Which of the following characterises you best?(If you are both a patient and a carer, please choo... = I am a carer*

PLEASE NOTE: if the patient you take care of is younger than 18 years old or legally incapacitated, please try to answer the questions in this survey from your perspective as their carer, being able to make decisions on their behalf.

Are you a member of a patient organisation?

- Yes
- No

**End of Block: Introductory questions to select target participants**

**Start of Block: Introductory questions**

Which country do you currently reside in?
 *(Please note that the survey is aimed at individuals currently living in a Member State of the European Union or in the United Kingdom, Switzerland, Norway, Iceland or Liechtenstein)*

- Austria
- Belgium
- Bulgaria
- Croatia
- Cyprus
- Czechia
- Denmark
- Estonia
- Finland
- France
- Germany
- Greece
- Hungary
- Iceland
- Ireland
- Italy
- Latvia
- Liechtenstein
- Lithuania
- Luxembourg
- Malta
- Netherlands
- Norway
- Poland
- Portugal
- Romania
- Slovakia
- Slovenia
- Spain
- Sweden
- Switzerland
- United Kingdom

What is your gender?

- Male
- Female
- Non-binary / other
- Prefer not to say

What is your age?
 *(Please note that participants should be at least 18 years old.)*

- 18-24
- 25-34
- 35-44
- 45-54
- 55-64
- 65-74
- 75 or older

*Display This Question:*

*If Which of the following characterises you best?(If you are both a patient and a carer, please choo... = I am a carer*

What is the age of the patient you take care of?

- Below 18
- 18-24
- 25-34
- 35-44
- 45-54
- 55-64
- 65-74
- 75 or older

What is the highest level of education that you have completed?

- Primary education (primary school)
- Secondary education (high school)
- Bachelor's degree or equivalent
- Master's degree or equivalent
- Doctorate degree

*Display This Question:*

*If Which of the following characterises you best?(If you are both a patient and a carer, please choo... = I am a patient*

Have you ever participated in a clinical trial or another type of medical study before?

 *A* ***clinical trial*** *is a medical study in which participants are assigned to receive an experimental treatment in order to investigate its effects on health outcomes, usually compared to another (or sometimes no) treatment.*

- Yes
- No
- I do not know

*Display This Question:*

*If Which of the following characterises you best?(If you are both a patient and a carer, please choo... = I am a carer*

Has the patient you take care of participated in a clinical trial or another type of medical study before?

 *A* ***clinical trial*** *is a medical study in which participants are assigned to receive an experimental treatment in order to investigate its effects on health outcomes, usually compared to another (or sometimes no) treatment.*

- Yes
- No
- I do not know

*Display This Question:*

*If Which of the following characterises you best?(If you are both a patient and a carer, please choo... = I am neither a patient nor a carer*

*And Are you a member of a patient organisation? = Yes*

As a member of a patient organisation, do you know any patients who have participated in a clinical trial or another type of medical study?

 *A* ***clinical trial*** *is a medical study in which participants are assigned to receive an experimental treatment in order to investigate its effects on health outcomes, usually compared to another (or sometimes no) treatment.*

- Yes
- No
- I am not sure

*Display This Question:*

*If Which of the following characterises you best?(If you are both a patient and a carer, please choo... = I am a patient*

Have you ever allowed your personal data, collected when receiving healthcare, to be re-used for medical research purposes?

 ***Personal data*** *is any information that is related to you, such as your name, gender, age, as well as information about your health (disease, blood test results) or your genetic data.* ***Pseudonymous data is also personal data.*** *Pseudonymous data is data that can no longer be attributed to a specific individual without the use of additional information, for instance key-coded data: e.g. a dataset where the names have been replaced with codes. On the other hand,* ***anonymised data is NOT personal data****: anonymised means that the individual is no longer identifiable.*

- Yes
- No
- I do not know

*Display This Question:*

*If Which of the following characterises you best?(If you are both a patient and a carer, please choo... = I am a carer*

Has the patient you take care of ever allowed their personal data, collected when receiving healthcare, to be re-used for medical research purposes? 

 ***Personal data*** *is any information that is related to you, such as your name, gender, age, as well as information about your health (disease, blood test results) or your genetic data.* ***Pseudonymous data is also personal data.*** *Pseudonymous data is data that can no longer be attributed to a specific individual without the use of additional information, for instance key-coded data: e.g. a dataset where the names have been replaced with codes. On the other hand,* ***anonymised data is NOT personal data****: anonymised means that the individual is no longer identifiable.*

- Yes
- No
- I do not know

*Display This Question:*

*If Which of the following characterises you best?(If you are both a patient and a carer, please choo... = I am neither a patient nor a carer*

*And Are you a member of a patient organisation? = Yes*

As a member of a patient organisation, do you know any patients who have ever allowed their personal data, collected when receiving healthcare, to be re-used for medical research purposes?

 ***Personal data****is any information that is related to you, such as your name, gender, age, as well as information about your health (disease, blood test results) or your genetic data.****Pseudonymous data is also personal data.****Pseudonymous data is data that can no longer be attributed to a specific individual without the use of additional information, for instance key-coded data: e.g. a dataset where the names have been replaced with codes. On the other hand,****anonymised data is NOT personal data****: anonymised means that the individual is no longer identifiable.*

- Yes
- No
- I do not know

*Display This Question:*

*If Which of the following characterises you best?(If you are both a patient and a carer, please choo... = I am a patient*

Have you followed any courses targeted towards patients on the topics of medicines development, clinical research or patient engagement?

- Yes
- No

*Display This Question:*

*If Which of the following characterises you best?(If you are both a patient and a carer, please choo... = I am a carer*

Have you followed any courses targeted towards carers on the topics of medicines development, clinical research or patient engagement?

- Yes
- No

*Display This Question:*

*If Which of the following characterises you best?(If you are both a patient and a carer, please choo... = I am neither a patient nor a carer*

*And Are you a member of a patient organisation? = Yes*

Have you followed any courses targeted towards members of patient organisations on the topics of medicines development, clinical research or patient engagement?

- Yes
- No

Which of the following disease areas do you have the most experience with *(multiple options possible)*?

- Cardiovascular diseases (e.g. cardiomyopathy)
- Diabetes (e.g. type 2 diabetes)
- Cancer (e.g. lung cancer)
- Gastrointestinal diseases (e.g. Crohn's disease)
- Respiratory diseases (e.g. chronic obstructive pulmonary disease)
- Urinary system diseases (e.g. chronic kidney disease)
- Metabolic diseases (e.g. Gaucher disease)
- Infectious diseases (e.g. COVID-19)
- Psychiatric disorders (e.g. bipolar disorder)
- Neurological disorders (e.g. multiple sclerosis)
- Rheumatic disorders (e.g. rheumatoid arthritis)
- Skin diseases (e.g. psoriasis)
- Gynecological disorders (e.g. endometriosis)
- Blood disorders (e.g. haemophilia)
- Autoimmune disorders (e.g. lupus)
- Rare diseases (e.g. cystic fibrosis)
- Other, please specify __________________________________________________

**End of Block: Introductory questions**

**Start of Block: GDPR and data control questions**

**Part A. Data protection and data control**

Have you ever heard of the EU General Data Protection Regulation (GDPR)?

- Yes
- No

*Display This Question:*

*If Have you ever heard of the EU General Data Protection Regulation (GDPR)? = Yes*

What is your main source of information about the GDPR? *(multiple options possible)*

- The news
- My employer
- Educational authorities (school, university)
- My friends and family
- Posts on social media (Facebook, Twitter)
- Notifications on websites and online applications (e.g., cookie notifications)
- Emails from companies that process my personal information (e.g., email from my bank)
- A healthcare provider (e.g., a hospital)
- Experience as participant in medical research (e.g., during the informed consent process)
- Specialised training on data protection
- Other, please specify __________________________________________________

Who do you think is responsible for the protection of personal data when the data is used for medical research? *(multiple options possible)*

- The patient whose personal data is used
- The data protection authority (DPA). *(DPAs are independent public authorities that monitor and supervise the application of the data protection law.)*
- The individual or entity that uses the personal data. *(For instance, the pharmaceutical company that sponsors the clinical trial in which the patient participates, or the hospital where the patient receives care.)*
- A patient organisation that is active in the disease area in which the research happens
- The research ethics committee that approved the study
- Other, please specify __________________________________________________
- I do not know

What rights do you think are guaranteed under the GDPR? *(multiple options possible)*

- Right to access my personal data collected online (e.g., through a health application)
- Right to access my personal data collected in a clinical trial or another study in which I participated
- Right to have my personal data deleted
- Right to transfer my personal data to another organisation
- Right to object to the use of my personal data (e.g., in a clinical trial for which I initially gave consent)
- Right that a human should always review decisions about my clinical care taken by an AI tool
- Right to financial compensation for sharing my personal data
- Right to inherit the personal data of a diseased relative
- I do not know

*Display This Question:*

*If Which of the following characterises you best?(If you are both a patient and a carer, please choo... = I am a patient*

*And Have you ever participated in a clinical trial or another type of medical study before?A clinical... = Yes*

Have you taken any of the following actions in the past two years? *(multiple options possible)*

- **I requested access to all my personal data** that was collected in the scope of a clinical trial or another medical research study
- **I objected to the use of my personal data** that was collected in the scope of a clinical trial or another medical research study
- **I withdrew consent for the use of my personal data** that was collected in the scope of a clinical trial or another medical research study
- **I requested that my personal data** that was collected in the scope of a clinical trial or another medical research study **was deleted**
- **I requested that my personal data** that was collected in the scope of a clinical trial or another medical research study **was transmitted to a different research center or healthcare provider**
- None of the above

*Display This Question:*

*If Which of the following characterises you best?(If you are both a patient and a carer, please choo... = I am a patient*

*And Have you ever participated in a clinical trial or another type of medical study before?A clinical... = No*

*Or If*

*Which of the following characterises you best?(If you are both a patient and a carer, please choo... = I am a patient*

*And Have you ever participated in a clinical trial or another type of medical study before?A clinical... = I do not know*

Have you taken any of the following actions in the past two years? *(multiple options possible)*

- **I requested access to all my personal data** that was collected in my health record, or through a smartphone health application/ medical device
- **I objected to the use of my personal data** that was collected in my health record, or through a smartphone health application/ medical device
- **I withdrew consent for the use of my personal data** that was collected in my health record, or through a smartphone health application/ medical device
- **I requested that my personal data** that was collected in my health record, or through a smartphone health application/ medical device **was deleted**
- **I requested that my personal data** that was collected in my health record, or through a smartphone health application/ medical device **was transmitted to a different healthcare provider or manufacturer**
- None of the above

*Display This Question:*

*If Which of the following characterises you best?(If you are both a patient and a carer, please choo... = I am a carer*

*And Has the patient you take care of participated in a clinical trial or another type of medical stud... = Yes*

Has the patient you take care of taken any of the following actions in the past two years? *(multiple options possible)*

- **Requested access to all their personal data** that was collected in the scope of a clinical trial or another medical research study
- **Objected to the use of their personal data** that was collected in the scope of a clinical trial or another medical research study
- **Withdrew consent for the use of their personal data** that was collected in the scope of a clinical trial or another medical research study
- **Requested that their personal data** that was collected in the scope of a clinical trial or another medical research study **was deleted**
- **Requested that their personal data** that was collected in the scope of a clinical trial or another medical research study **was transmitted to a different research center or healthcare provider**
- None of the above

*Display This Question:*

*If Which of the following characterises you best?(If you are both a patient and a carer, please choo... = I am a carer*

*And Has the patient you take care of participated in a clinical trial or another type of medical stud... = No*

*Or If*

*Which of the following characterises you best?(If you are both a patient and a carer, please choo... = I am a carer*

*And Has the patient you take care of participated in a clinical trial or another type of medical stud... = I do not know*

Has the patient you take care of taken any of the following actions in the past two years? *(multiple options possible)*

- **Requested access to all their personal data** that was collected in their health record, or through a smartphone health application/ medical device
- **Objected to the use of their personal data** that was collected in their health record, or through a smartphone health application/ medical device
- **Withdrew consent for the use of their personal data** that was collected in their health record, or through a smartphone health application/ medical device
- **Requested that their personal data** that was collected in their health record, or through a smartphone health application/ medical device **was deleted**
- **Requested that their personal data** that was collected in their health record, or through a smartphone health application/ medical device **was transmitted to a different healthcare provider or manufacturer**
- None of the above

*Display This Question:*

*If Which of the following characterises you best?(If you are both a patient and a carer, please choo... = I am a patient*

*And Have you ever participated in a clinical trial or another type of medical study before?A clinical... = Yes*

Do you see yourself using any of the following rights in the next year? *(multiple options possible)*

- **To request access to my personal data** that was collected in the scope of a clinical trial or another medical research study
- **To object to the use of my personal data** that was collected in the scope of a clinical trial or another medical research study
- **To withdraw consent for the use of my personal data** that was collected in the scope of a clinical trial or another medical research study
- **To request that my personal data** that was collected in the scope of a clinical trial or another medical research study **is deleted**
- **To request that my personal data** that was collected in the scope of a clinical trial or another medical research study **is transmitted to a different research center or healthcare provider**
- None of the above

*Display This Question:*

*If Which of the following characterises you best?(If you are both a patient and a carer, please choo... = I am a patient*

*And Have you ever participated in a clinical trial or another type of medical study before?A clinical... = No*

*Or If*

*Which of the following characterises you best?(If you are both a patient and a carer, please choo... = I am a patient*

*And Have you ever participated in a clinical trial or another type of medical study before?A clinical... = I do not know*

Do you see yourself using any of the following rights in the next year? *(multiple options possible)*

- **To request access to all my personal data** that was collected in my health record, or through a smartphone health application/ medical device
- **To object to the use of my personal data** that was collected in my health record, or through a smartphone health application/ medical device
- **To withdraw consent for the use of my personal data** that was collected in my health record, or through a smartphone health application/ medical device
- **To request that my personal data** that was collected in my health record, or through a smartphone health application/ medical device **is deleted**
- **To request that my personal data** that was collected in my health record, or through a smartphone health application/ medical device **is transmitted to a different healthcare provider or manufacturer**
- None of the above

*Display This Question:*

*If Which of the following characterises you best?(If you are both a patient and a carer, please choo... = I am a carer*

*And Has the patient you take care of participated in a clinical trial or another type of medical stud... = Yes*

Do you see the patient you take care of using any of the following rights in the next year? *(multiple options possible)*

- **To request access to their personal data** that was collected in the scope of a clinical trial or another medical research study
- **To object to the use of their personal data** that was collected in the scope of a clinical trial or another medical research study
- **To withdraw consent for the use of their personal data** that was collected in the scope of a clinical trial or another medical research study
- **To request that their personal data** that was collected in the scope of a clinical trial or another medical research study **is deleted**
- **To request that their personal data** that was collected in the scope of a clinical trial or another medical research study **is transmitted to a different research center or healthcare provider**
- None of the above

*Display This Question:*

*If Which of the following characterises you best?(If you are both a patient and a carer, please choo... = I am a carer*

*And Has the patient you take care of participated in a clinical trial or another type of medical stud... = No*

*Or If*

*Which of the following characterises you best?(If you are both a patient and a carer, please choo... = I am a carer*

*And Has the patient you take care of participated in a clinical trial or another type of medical stud... = I do not know*

Do you see the patient you take care of using any of the following rights in the next year? *(multiple options possible)*

- **To request access to their personal data** that was collected in their health record, or through a smartphone health application/ medical device
- **To object to the use of their personal data** that was collected in their health record, or through a smartphone health application/ medical device
- **To withdraw consent for the use of their personal data** that was collected in their health record, or through a smartphone health application/ medical device
- **To request that their personal data** that was collected in their health record, or through a smartphone health application/ medical device **is deleted**
- **To request that their personal data** that was collected in their health record, or through a smartphone health application/ medical device **is transmitted to a different healthcare provider or manufacturer**
- None of the above

*Display This Question:*

*If Which of the following characterises you best?(If you are both a patient and a carer, please choo... = I am neither a patient nor a carer*

*And Are you a member of a patient organisation? = Yes*

*And As a member of a patient organisation, do you know any patients who have participated in a clinic... = Yes*

| 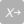 |
| --- |

Based on your experience as a member of a patient organisation, how often have patients you know taken any of the following actions in the past two years?

|  | Never | Sometimes | About half the time | Most of the time | Always | I do not know |
| --- | --- | --- | --- | --- | --- | --- |
| **Requested access to all their personal data** that was collected in the scope of a clinical trial or another medical research study |  |  |  |  |  |  |
| **Objected to the use of their personal data** that was collected in the scope of a clinical trial or another medical research study |  |  |  |  |  |  |
| **Withdrew consent for the use of their personal data** that was collected in the scope of a clinical trial or another medical research study |  |  |  |  |  |  |
| **Requested that their personal data** that was collected in the scope of a clinical trial or another medical research study **was deleted** |  |  |  |  |  |  |
| **Requested that their personal data** that was collected in the scope of a clinical trial or another medical research study **was transmitted to a different research center or healthcare provider** |  |  |  |  |  |  |

*Display This Question:*

*If Which of the following characterises you best?(If you are both a patient and a carer, please choo... = I am neither a patient nor a carer*

*And Are you a member of a patient organisation? = Yes*

*And As a member of a patient organisation, do you know any patients who have participated in a clinic... = No*

*Or If*

*Which of the following characterises you best?(If you are both a patient and a carer, please choo... = I am neither a patient nor a carer*

*And Are you a member of a patient organisation? = Yes*

*And As a member of a patient organisation, do you know any patients who have participated in a clinic... = I am not sure*

Based on your experience as a member of a patient organisation, how often have patients you know taken any of the following actions in the past two years?

|  | Never | Sometimes | About half the time | Most of the time | Always | I do not know |
| --- | --- | --- | --- | --- | --- | --- |
| **Requested access to all their personal data** that was collected in their health record, or through a smartphone health application/ medical device |  |  |  |  |  |  |
| **Objected to the use of their personal data** that was collected in their health record, or through a smartphone health application/ medical device |  |  |  |  |  |  |
| **Withdrew consent for the use of their personal data** that was collected in their health record, or through a smartphone health application/ medical device |  |  |  |  |  |  |
| **Requested that their personal data** that was collected in their health record, or through a smartphone health application/ medical device **was deleted** |  |  |  |  |  |  |
| **Requested that their personal data** that was collected in their health record, or through a smartphone health application/ medical device **was transmitted to a different healthcare provider or manufacturer** |  |  |  |  |  |  |

*Display This Question:*

*If Which of the following characterises you best?(If you are both a patient and a carer, please choo... = I am neither a patient nor a carer*

*And Are you a member of a patient organisation? = Yes*

*And As a member of a patient organisation, do you know any patients who have participated in a clinic... = Yes*

| 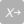 |
| --- |

Based on your experience as a member of a patient organisation, how likely do you think it is that patients you know take any of the actions described above in the next one year?

- Very unlikely
- Not likely
- Neutral
- Likely
- Very likely
- I do not know

*Display This Question:*

*If Which of the following characterises you best?(If you are both a patient and a carer, please choo... = I am neither a patient nor a carer*

*And Are you a member of a patient organisation? = Yes*

*And As a member of a patient organisation, do you know any patients who have participated in a clinic... = No*

*Or If*

*Which of the following characterises you best?(If you are both a patient and a carer, please choo... = I am neither a patient nor a carer*

*And Are you a member of a patient organisation? = Yes*

*And As a member of a patient organisation, do you know any patients who have participated in a clinic... = I am not sure*

Based on your experience as a member of a patient organisation, how likely do you think it is that patients you know take any of the actions described above in the next one year?

- Very unlikely
- Not likely
- Neutral
- Likely
- Very likely
- I do not know

| Page Break |  |
| --- | --- |

What does control over the use of your personal data (also called data control) mean according to you? *(open question)*

________________________________________________________________

| 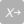 |
| --- |

Below, you can find a list of tools to exercise control over your personal data when it is used for medical research. Please indicate how important the use of each element is to you.
 *(If you are a carer, or a member of a patient organisation who is not a patient/carer, try to answer this question by taking the perspective of the patient you take care of or the patients you know in the disease area you are active in)*

|  | Not at all important | Slightly important | Moderately important | Very important | Extremely important |
| --- | --- | --- | --- | --- | --- |
| **INDIVIDUALLY PROVIDED INFORMATION (Primary use):**I am individually informed about the use of my personal data when the data is used in a medical research study |  |  |  |  |  |
| **INDIVIDUALLY PROVIDED INFORMATION (Secondary use /  Re-use)**: I am individually informed about the re-use of my personal data in EACH subsequent different medical research study performed by the original or a new researcher |  |  |  |  |  |
| **CONSENT (Primary use)**: I am asked to consent for the use of my personal data in a medical research study |  |  |  |  |  |
| **CONSENT (Secondary use / Re-use)**: I am asked to consent for EACH subsequent re-use of my personal data in different medical research studies by the original or a new researcher |  |  |  |  |  |
| **ACCESS**: I can access my personal data and receive a copy of it |  |  |  |  |  |
| **SHARING**: I can decide with whom my personal data is further shared (in case my data is already used by an individual or a company) |  |  |  |  |  |
| **PORTABILITY**: I can ask that my personal data is transmitted from one individual/company/hospital to another individual/company/hospital |  |  |  |  |  |
| **USE**: I can control for what purposes my personal data is used (e.g., through an online platform) |  |  |  |  |  |
| **DELETION**: I can ask that my personal data is deleted |  |  |  |  |  |
| **COLLECTIVE CONTROL**: I can delegate my individual control (in the form of any of the tools described above) to an entity that I trust (such as a specialised organisation: patient organisation, data intermediary, data altruism organisation, or other) |  |  |  |  |  |

*If you find that one or more elements are missing from the list presented to you above, you can add them here. Please also add a short description of the element(s).*

________________________________________________________________

*Display This Question:*

*If Which of the following characterises you best?(If you are both a patient and a carer, please choo... = I am a patient*

What **positive outcomes** do you think individual control over your personal data can have **for you**? *(open question)*

________________________________________________________________

*Display This Question:*

*If Which of the following characterises you best?(If you are both a patient and a carer, please choo... = I am a carer*

What **positive outcomes** do you think individual control over their personal data can have **for the patient you take care of?** *(open question)*

________________________________________________________________

*Display This Question:*

*If Which of the following characterises you best?(If you are both a patient and a carer, please choo... = I am neither a patient nor a carer*

*And Are you a member of a patient organisation? = Yes*

What **positive outcomes** do you think individual control over their personal data can have **for patients in your disease area?** *(open question)*

________________________________________________________________

What **positive outcomes** do you think having individual control over personal data can have **for medical research?** *(open question)*

________________________________________________________________

*Display This Question:*

*If Which of the following characterises you best?(If you are both a patient and a carer, please choo... = I am a patient*

Do you think individual control over your personal data may have **any undesirable effects on you**?

- Yes (please explain) __________________________________________________
- No

*Display This Question:*

*If Which of the following characterises you best?(If you are both a patient and a carer, please choo... = I am a carer*

Do you think individual control over their personal data may have **any undesirable effects on the patient you take care of?**

- Yes (please explain) __________________________________________________
- No

*Display This Question:*

*If Which of the following characterises you best?(If you are both a patient and a carer, please choo... = I am neither a patient nor a carer*

*And Are you a member of a patient organisation? = Yes*

Do you think individual control over their personal data may have **any undesirable effects on patients in your disease area?**

- Yes (please explain) __________________________________________________
- No

Do you think having individual control over personal data may have **any undesirable effects for medical research?**

- Yes (please explain) __________________________________________________
- No

| Page Break |  |
| --- | --- |

*Display This Question:*

*If Which of the following characterises you best?(If you are both a patient and a carer, please choo... = I am a patient*

| 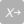 |
| --- |

**Data altruism without patient involvement:** Imagine that you can consent that your personal data is shared with a so-called data altruism organisation. This data altruism organisation will, in turn, have the right to share your data with other users (individual researchers, companies, public bodies, etc) as long as these users intend to use the data for medical research. You can withdraw your consent at any time. You will not be informed about each individual use of your data.  

 **How likely are you to share your personal data** with this data altruism organisation?

- Very unlikely
- Not likely
- Neutral
- Likely
- Very likely

*Display This Question:*

*If Which of the following characterises you best?(If you are both a patient and a carer, please choo... = I am a carer*

| 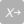 |
| --- |

**Data altruism without patient involvement:** Imagine that the patient you take care of can consent that his/her personal data is shared with a so-called data altruism organisation. This data altruism organisation will, in turn, have the right to share the patient's data with other users (individual researchers, companies, public bodies, etc) as long as these users intend to use the data for medical research. The patient you take care of can withdraw consent at any time. He/she will not be informed about each individual use of his/her data.  

 **How likely would the patient you take care of be to share his/her personal data** with this data altruism organisation?

- Very unlikely
- Not likely
- Neutral
- Likely
- Very likely

*Display This Question:*

*If Which of the following characterises you best?(If you are both a patient and a carer, please choo... = I am neither a patient nor a carer*

*And Are you a member of a patient organisation? = Yes*

| 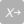 |
| --- |

**Data altruism without patient involvement (your perspective as a member of a patient organisation):** Imagine that patients in your disease area can consent that their personal data is shared with a so-called data altruism organisation. This data altruism organisation will, in turn, have the right to share the patients' data with other users (individual researchers, companies, public bodies, etc) as long as these users intend to use the data for medical research. The patients can withdraw consent at any time. They will not be informed about each individual use of their data.  

 **How likely would they be to share their personal data** with this data altruism organisation?

- Very unlikely
- Not likely
- Neutral
- Likely
- Very likely

*Display This Question:*

*If Which of the following characterises you best?(If you are both a patient and a carer, please choo... = I am a patient*

| 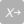 |
| --- |

**Data altruism with patient involvement**: Imagine the same scenario as described above, but in this case, there are patient representatives involved in the data altruism organisation. Each time the data altruism organisation has to make a decision whether they share data or not, the patient representatives are meaningfully involved in the decision-making process.

 **How likely are you to share your personal data** with the data altruism organisation?

- Very unlikely
- Not likely
- Neutral
- Likely
- Very likely

*Display This Question:*

*If Which of the following characterises you best?(If you are both a patient and a carer, please choo... = I am a carer*

| 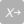 |
| --- |

**Data altruism with patient involvement**: Imagine the same scenario as described above, but in this case, there are patient representatives involved in the data altruism organisation. Each time the data altruism organisation has to make a decision whether they share data or not, the patient representatives are meaningfully involved in the decision-making process.

 **How likely would the patient you take care of be to share his/her personal data** with the data altruism organisation?

- Very unlikely
- Not likely
- Neutral
- Likely
- Very likely

*Display This Question:*

*If Which of the following characterises you best?(If you are both a patient and a carer, please choo... = I am neither a patient nor a carer*

*And Are you a member of a patient organisation? = Yes*

| 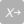 |
| --- |

**Data altruism with patient involvement (your perspective as a member of a patient organisation)**: Imagine the same scenario as described above, but in this case, there are patient representatives involved in the data altruism organisation. Each time the data altruism organisation has to make a decision whether they share data or not, the patient representatives are meaningfully involved in the decision-making process.

 **How likely would patients in your disease area be to share their personal data** with the data altruism organisation?

- Very unlikely
- Not likely
- Neutral
- Likely
- Very likely

*Display This Question:*

*If Which of the following characterises you best?(If you are both a patient and a carer, please choo... = I am a patient*

How would you ideally like to be informed about how your personal data is being used in medical research?

- Via my electronic health record
- Via an EU portal dedicated to medical research
- Via a personalised email newsletter
- Via a smartphone application
- I do not need to receive information about the projects for which my personal data is used
- Other, please specify __________________________________________________
- No opinion

*Display This Question:*

*If Which of the following characterises you best?(If you are both a patient and a carer, please choo... = I am a carer*

How do you think the patient you take care of would ideally like to be informed about how their personal data is being used in medical research?

- Via their electronic health record
- Via an EU portal dedicated to medical research
- Via a personalised email newsletter
- Via a smartphone application
- They do not need to receive information about the projects for which their personal data is used
- Other, please specify __________________________________________________
- No opinion

*Display This Question:*

*If Which of the following characterises you best?(If you are both a patient and a carer, please choo... = I am neither a patient nor a carer*

*And Are you a member of a patient organisation? = Yes*

As a member of a patient organisation, how do you think patients in your disease area would ideally like to be informed about how their personal data is being used in medical research?

- Via their electronic health record
- Via an EU portal dedicated to medical research
- Via a personalised email newsletter
- Via a smartphone application
- They do not need to receive information about the projects for which their personal data is used
- Other, please specify __________________________________________________
- No opinion

If you have **any additional comments** regarding data control and the use of personal data for medical research, please feel free to leave them in the text box below:

________________________________________________________________

If you would like **to participate in future research (for instance interviews)**that will explore in more detail the issues related to data control and data protection, **please share your email address below**:

________________________________________________________________

**End of Block: GDPR and data control questions**
